# Computational Analysis of Deleterious SNPs in *NRAS* to Assess Their Potential Correlation With Carcinogenesis

**DOI:** 10.3389/fgene.2022.872845

**Published:** 2022-08-16

**Authors:** Mohammed Y. Behairy, Mohamed A. Soltan, Mohamed S. Adam, Ahmed M. Refaat, Ehab M. Ezz, Sarah Albogami, Eman Fayad, Fayez Althobaiti, Ahmed M. Gouda, Ashraf E. Sileem, Mahmoud A. Elfaky, Khaled M. Darwish, Muhammad Alaa Eldeen

**Affiliations:** ^1^ Department of Microbiology and Immunology, Faculty of Pharmacy, University of Sadat City, Sadat City, Egypt; ^2^ Department of Microbiology and Immunology, Faculty of Pharmacy, Sinai University, Ismailia, Egypt; ^3^ Department of Pharmacology, Faculty of Pharmacy, Suez Canal University, Ismailia, Egypt; ^4^ Zoology Departmen, Faculty of Science, Minia University, El-Minia, Egypt; ^5^ Department of Pharmacology, Faculty of Medicine, University of Khartoum, Khartoum, Sudan; ^6^ Department of Biotechnology, College of Sciences, Taif University, Taif, Saudi Arabia; ^7^ Department of Pharmacy Practice, Faculty of Pharmacy, Zagazig University, Zagazig, Egypt; ^8^ Department of Chest Diseases, Faculty of Medicine, Zagazig University, Zagazig, Egypt; ^9^ Department of Natural Products, Faculty of Pharmacy, King Abdulaziz University, Jeddah, Saudi Arabia; ^10^ Centre for Artificial Intelligence in Precision Medicines, King Abdulaziz University, Jeddah, Saudi Arabia; ^11^ Department of Medicinal Chemistry, Faculty of Pharmacy, Suez Canal University, Ismailia, Egypt; ^12^ Cell Biology, Histology and Genetics Division, Zoology Department, Faculty of Science, Zagazig University, Zagazig, Egypt

**Keywords:** *NRAS* gene, single nucleotide polymorphism, computational analysis, carcinogenesis, precision medicine

## Abstract

The *NRAS* gene is a well-known oncogene that acts as a major player in carcinogenesis. Mutations in the *NRAS* gene have been linked to multiple types of human tumors. Therefore, the identification of the most deleterious single nucleotide polymorphisms (SNPs) in the *NRAS* gene is necessary to understand the key factors of tumor pathogenesis and therapy. We aimed to retrieve *NRAS* missense SNPs and analyze them comprehensively using sequence and structure approaches to determine the most deleterious SNPs that could increase the risk of carcinogenesis. We also adopted structural biology methods and docking tools to investigate the behavior of the filtered SNPs. After retrieving missense SNPs and analyzing them using six *in silico* tools, 17 mutations were found to be the most deleterious mutations in *NRAS*. All SNPs except S145L were found to decrease *NRAS* stability, and all SNPs were found on highly conserved residues and important functional domains, except R164C. In addition, all mutations except G60E and S145L showed a higher binding affinity to GTP, implicating an increase in malignancy tendency. As a consequence, all other 14 mutations were expected to increase the risk of carcinogenesis, with 5 mutations (G13R, G13C, G13V, P34R, and V152F) expected to have the highest risk. Thermodynamic stability was ensured for these SNP models through molecular dynamics simulation based on trajectory analysis. Free binding affinity toward the natural substrate, GTP, was higher for these models as compared to the native NRAS protein. The Gly13 SNP proteins depict a differential conformational state that could favor nucleotide exchange and catalytic potentiality. A further application of experimental methods with all these 14 mutations could reveal new insights into the pathogenesis and management of different types of tumors.

## Introduction

Single nucleotide polymorphism (SNP) is defined as a variation in one base in a DNA nucleotide that happens at a specific site of the studied genome (Vignal et al., 2002). In the human genome, SNPs represent the most stable and abundant form of genetic variation (Liao and Lee, 2010). In addition, a high rate of SNP occurrence in a gene represented a biological marker that this specific gene can be correlated with important traits (Yeam, 2016). There are different forms of SNPs, and missense SNP, which is a type of non-synonymous SNP (nsSNP) substitution characterized by amino acid substitution with the possibility of forming a mutated protein with new structural and functional features, is the most important form of SNPs, which may lead to a significant alteration in the progression of different diseases (Emadi et al., 2020). This form of SNP can produce deleterious actions by minimizing protein solubility, destabilizing protein tertiary structure, and manipulating gene regulation through modifying transcription regulatory proteins (Chasman and Adams, 2001). Moreover, this form of SNP can modify the cell fitness with an increased growth preference and finally result in tumorigenesis (Tan, 2017). For this purpose, several studies are currently highly concerned with the pathogenic consequences of these SNPs, and many trials have tried to correlate them with changeable effects on humans (Dakal et al., 2017). While a large number of missense SNPs for several genes has been reported and deposited in databases, the whole number of missense SNPs for each gene showed a variation in the attribution to disease modification; therefore, it became essential to filter the SNPs with possible pathogenicity from the pool of neutral variants (Zhang et al., 2020). Although clinical investigation with wet-lab experiments would provide the most accurate approach for the estimation of the consequences of each SNP, this process is costly and very time-consuming (Jia et al., 2014). As an alternative, computational methods offer an excellent choice for researchers with the advantages of being an affordable and time-saving approach (Soltan et al., 2021). The application of this computational approach would result in a shortlist of possible deleterious SNPs, which could be investigated clinically (Patnala et al., 2013). Furthermore, the continuous improvement in the field of structural biology and its related computational tools made it possible to analyze the structural consequences of filtered SNPs and predict the functional alteration of the analyzed protein as a result of the structural modification (Rajput and Gahlay, 2021).

The Ras genes, *HRAS*, *KRAS*, and *NRAS*, are members of the Ras family with GTPase activity. These genes function as molecular switches in the cell, where Ras GTP represents the active state, while Ras GDP represents the inactive state (Damani Shah et al., 2019). The function of Ras proteins in the regulation of cellular signal pathways has attracted the attention of numerous researchers in the last few decades (Muñoz-Maldonado et al., 2019). Studies have shown that these proteins have significant roles in regulating cell motility (Voice et al., 1999), controlling cell apoptosis (Bivona et al., 2006), and organizing cell proliferation (Matallanas et al., 2006). The distinct role of each Ras member can be correlated with various post-translational modifications (PTMs) at the C-termini, where each protein localizes a different subcellular membrane based on its modification and subsequently stimulates a specific signaling pathway (Rocks et al., 2006). While the plasma membrane is considered the basic site for all the Ras proteins, further investigations proved that they can also localize in the endoplasmic reticulum, Golgi apparatus, and mitochondria (Chiu et al., 2002; Fehrenbacher et al., 2009).

The *NRAS* gene is one of the most studied oncogenes as it acts as a major player in carcinogenesis (Eisfeld et al., 2014). Several studies have attributed mutations in the *NRAS* gene to numerous forms of human tumors, and it was reported that variants of *NRAS* differ greatly in their downstream effects and consequently participate in cell transformation to a malignant state (Yin et al., 2019). For example, deleterious SNPs in *NRAS* were analyzed for their role in the progression of retinoblastoma (Sun et al., 2019), promotion of lung metastasis (Giannou et al., 2017), and induction of melanoma (Kwong et al., 2012). In the current time of personalized medicine development, it is highly recommended that deep analysis of the cancer genomic landscape is an essential step to fight against cancer, the first cause of human death (Gharib et al., 2021).

Hence, the aim of the current study is to retrieve *NRAS* missense SNPs and filter them to determine the most deleterious SNPs using computational tools. Moreover, structural biology and docking tools are employed to investigate the behavior of the filtered SNPs and their modified roles in the cellular environment. Altogether, these data would contribute significantly to the field of personalized medicine against several forms of tumors.

## Materials and Methods

We depended on the National Center for Biotechnology Information (NCBI) databases besides Ensembl databases for retrieving general information regarding the *NRAS* gene. In addition, we used the Genecards database (Genecards.org) to retrieve gene ontology data and (compartments.jensenlab.org) as the source of data regarding the subcellular localization.

### Retrieving *NRAS* Gene Variants

NCBI was used to retrieve *NRAS* gene variants using variation viewer and by selecting dbSNP as the source database (https://www.ncbi.nlm.nih.gov/variation/view/). “NRAS” or 4,893 [geneid] was used as an entry. We filtered those retrieved SNPs and selected only the missense variants for further analysis and screening.

### Predicting Deleterious Variants Using Various *In Silico* Tools

We used six different *in silico* tools for predicting the most deleterious variants on the function of NRAS protein: SIFT (Sorting Intolerant from Tolerant), PolyPhen (Polymorphism Phenotyping), PROVEAN (Protein Variation Effect Analyzer), SNAP, SNP&GO, and PHD-SNP. The SNPs that were predicted to be deleterious by all tools were considered the most deleterious ones. Using six different tools with varying algorithms, an approach that was applied previously (Hossain et al., 2020; Dhar et al., 2022; Rajput and Gahlay, 2021), could increase the confidence and accuracy of our analysis.

The SIFT server relies on a sequence homology approach in addition to the properties of residues to analyze the probability of developing harmful impacts with the missense variants (https://sift.bii.a-star.edu.sg/) (Sim et al., 2012). PolyPhen-2 analyzes the impacts of amino acid substitutions based on physical considerations in addition to comparative methods (http://genetics.bwh.harvard.edu/pph2) (Adzhubei et al., 2010). The PROVEAN tool uses fast methods to compute the scores of pairwise alignment and calculates the required prediction of the impact of the amino acid substitutions (http://provean.jcvi.org/seq_submit.php) (Choi and Chan, 2015). The SNPs&GO tool depends on the functional annotation of the analyzed proteins to identify the deleterious gene variants (https://snps.biofold.org/snpsand-go/snps-and-go.html) (Capriotti et al., 2013). The PHD-SNP server uses support vector machines for analyzing the association between the resulting phenotypes due to amino acid substitution and the development of human diseases (http://snps.biofold.org/phd-snp/phd-snp.html) (Capriotti et al., 2006). SNAP2 has a novel neural network that enables it to identify the effect of SNPs and the neutral SNPs (https://rostlab.org/services/snap/) (Hecht et al., 2015).

### Predicting Single Nucleotide Polymorphism Impact on NRAS Protein Stability

SNPs’ impact on NRAS protein stability was analyzed using both the I-Mutant 2.0 server and the Mu-Pro tool. The I-Mutant 2.0 server (https://folding.biofold.org/i-mutant/i-mutant2.0.html) relies on a support vector machine for the prediction of the direction and the value of the free energy change (DDG) (Capriotti et al., 2005). I-Mutant 2.0 was tested on the ProTherm database, which comprised the largest experimental information about the change in free energy related to the stability of proteins with mutations (Bava et al., 2004). The Mu-Pro tool (http://mupro.proteomics.ics.uci.edu/) provides a support vector machines method that was tested and cross-validated with an accuracy of 84% (Cheng et al., 2006).

### Identifying Single Nucleotide Polymorphism Locations on NRAS Domains

We used InterPro to determine the locations of the most deleterious variants on the domains of NRAS protein (https://www.ebi.ac.uk/interpro/). InterPro could perform the functional analysis related to the nominated protein and determine its functional domains and important motifs (Blum et al., 2021).

### Identifying Phylogenetically Conserved Residues in NRAS Protein

We used the ConSurf bioinformatics tool to analyze the evolutionary conservation of the residues of NRAS protein (https://consurf.tau.ac.il). ConSurf can analyze the phylogenetic relationships that exist between various homologous sequences to perform the needed analysis with the calculation of the conservation score that ranges from one to nine. Furthermore, the structural and functional residues are identified as well (Berezin et al., 2004; Ashkenazy et al., 2016).

### Analyzing Secondary Structures of NRAS Protein

We used the SOPMA server (https://npsa-prabi.ibcp.fr/cgi-bin/npsa_automat.pl?page=/NPSA/npsa_sopma.html) for identifying the predicted secondary structures of NRAS protein and the alignment of the mutant amino acids in these secondary structures. The SOPMA server can predict the secondary structures of a selected protein by analyzing the multiple alignments of this protein’s sequence (Geourjon and Deléage, 1995).

### Tertiary Structure Prediction and Validation of Proteins With Filtered Single Nucleotide Polymorphisms

The filtration step of missense SNPs resulted in 17 SNPs. The 3D structures of these filtered SNPs were predicted using SWISS-MODEL, a fully automated protein homology modeling server (Waterhouse et al., 2018). This server performs a fully automated prediction assessment, relying on the continuously deposited protein tertiary structures in the protein data bank. Regarding the *NRAS* gene, available data on UniProt demonstrated that the site 166–185 represents a hypervariable region, and for this purpose, the sequence 1–165 was uploaded to SWISS-MODEL to select our template for the tertiary structure prediction of 17 mutated proteins with the filtered SNPs. Following structure prediction, the generated models were further validated through Ramachandran plot analysis, which was integrated as an assessment tool in the SWISS-MODEL server, in addition to the ProSA webserver (Wiederstein and Sippl, 2007), which was also utilized for the validation process. Moreover, the TM-Align web server (https://zhanglab.ccmb.med.umich.edu/TM-align/) was employed to estimate the TM-score and root-mean-square deviation (RMSD) values of the parent model selected by SWISS-MODEL and the mutant ones that were predicted. The TM-score gives information about the degree of similarity between submitted structures, and its value ranges between 0 and 1 (a value of 1 represents a complete match between analyzed structures). On the other hand, the RMSD value indicates the average distance between alpha-carbon backbones of analyzed models, and the higher this value is, the more deviation is predicted between analyzed molecules (Zhang and Skolnick, 2005).

### Assessment of Mutated Proteins Through Molecular Docking-Coupled Molecular Dynamics

To estimate the consequences of filtered SNPs on the cellular behavior of NRAS protein, a molecular docking study between NRAS (the wild-type and the predicted mutated models) and GTP molecule (the ligand of NRAS active state) was proposed. As mentioned in the introduction section, NRAS is a GTP-binding protein that is switched on by incoming signals, leading to a turn-on for genes related to cell growth and differentiation. Therefore, SNPs in *NRAS* may stimulate a permanently activated form (NRAS-GTP), leading to overactive cellular signaling and ultimately cancer. The NRAS wild tertiary structure (PDB ID: 5UHV) was downloaded in PDB format to act as a control receptor for the current docking study; the deposited molecules in the receptor were first removed and the generated molecule saved to act as a control for its binding score with GTP to validate the docking scores of GTP-binding with other mutated models. The docking was performed through AutoDock Vina (Oleg and Arthur, 2010), the 3D conformation of the docked complexes was visualized by the molecular graphics system PyMOL (Seeliger and De Groot, 2010), and a 2D chart of the interacting residues for the generated complexes was investigated by LIGPLOT (Wallace et al., 1995).

Docked GTPase NRAS models were subjected to molecular dynamics simulations using GROMACS-5.1.4, CHARMM36m, and CHARMM-General forcefields within the TIP3P water solvation model and under periodic boundary conditions (Páll et al., 2015). Protein ionization was set at physiological pH 7.4, and system neutralization was done via chloride and potassium ions. Steepest-descent algorithm-minimization steps were performed at 5 ps, followed by equilibration at the initial NVT ensemble (Berendsen-temp method; 100 ps at 303.15 K) and final NPT ensemble (Parrinello-Rahmann barostat; 100 ps at one atmospheric pressure and 303.15 K) (Elhady et al., 2021). Molecular dynamics were run for 100 ns under NPT ensemble and Particle-Mesh-Ewald algorithms for computing long-range electrostatic interaction. Trajectory analysis was performed using RMSDs (Å), RMS fluctuations (RMSFs; Å), gyration radii (Rg; Å), and solvent-accessible surface area (SASA; nm^2^). Free binding energies for GTP substrate binding to GTPase NRAS were estimated using Molecular Mechanics/Poisson-Boltzmann (MM-PBSA; kJ/mol) single trajectory calculations (Kumari et al., 2014b). Visual Molecular Dynamics (VMD V.1.9.3) software (Illinois University, Urbana-Champaign, United States) was used for hydrogen bond analysis. Hydrogen bond distance/angle cut-offs were defined at 3.0 Å/20°. Conformational analysis and visualization of the simulated complexes at specified timeframes were performed using the PyMOL software.

### Normal Mode Analyses Through Torsional Network Model

The collective flexibilities/motion functions of the constructed GTPase NRAS were assessed using the iMODS online server (http://www.imods.chaconlab.org/) by applying the elastic torsional network model, whose degrees of freedom are the protein backbone torsion angles (López-Blanco et al., 2014). This approach is fast and accurate while being capable of assessing the collective protein’s motion and inherited dynamics based on normal-state analyses of respective internal dihedral angles and torsional coordinates. Furthermore, this server can predict several parameters reflecting structural flexibility/deformation reflecting significant deviation from the normal distribution values obtained from thousands of deposited reference sets.

### Identifying Single Nucleotide Polymorphism Impact on NRAS Protein Structure

We used the HOPE server (https://www3.cmbi.umcn.nl/hope/) for analyzing the SNPs’ impact on the NRAS protein 3D structure. The HOPE server depends on several sources for collecting the needed information in addition to building homology models using the YASARA program to perform the required function (Venselaar et al., 2010).

### Predicting the Sites of Post-Translational Modification

The MusiteDeep server (https://www.musite.net) was used to predict the sites of different types of PTMs. PTMs have a major role in regulating the function of proteins; thus, the identification of PTMs is important in analyzing disease pathogenesis (Wang et al., 2017; Wang et al., 2019; Wang et al., 2020).

### Gene–Gene Interaction Analysis

We used the GeneMANIA tool (http://www.genemania.org) for generating the gene–gene interaction network of the *NRAS* gene. The GeneMANIA tool could predict the strongly interacted genes with a selected gene using various sorts of data and resources: co-expression information, physical interaction information, co-localization data, functional relationships, information regarding pathways, genetic interaction data, and data regarding protein domains (Warde-Farley et al., 2010).

## Results

The workflow of the analysis steps applied in the current study is illustrated in Supplementary Figure S1.

### General Information

The *NRAS* gene (NCBI Gene ID: 4893) is a protein-coding gene that is located at 1p13.2. It comprises seven exons and has a length of 12,303 nucleotides; it has one transcript (ensemble.org). The membrane protein encoded by this gene shuttles between the plasma membrane and the Golgi apparatus and is characterized by GTPase activity (https://www.ncbi.nlm.nih.gov/gene/4893). The subcellular localization of the *NRAS* gene is illustrated in Supplementary Figure S2A (Compartments.jensenlab.org/), and its gene ontology is shown in Supplementary Figure S2B (Genecards.org).

### Retrieving *NRAS* Gene Variants

In the *NRAS* gene, 4,542 single nucleotide variations were found (accessed 31 December 2021). Among these SNPs, there were 113 missense SNPs, 62 synonymous SNPs, 73 5′ untranslated region (UTR) variants, 1,405 3′ UTR variants, and 2,435 intron SNPs, in addition to the other downstream and upstream SNPs.

### Determination of Deleterious Variants

Six different bioinformatics tools (SIFT, PolyPhen-2, PROVEAN, SNAP, SNP&GO, and PHD-SNP) were used to determine the deleterious variants in the *NRAS* gene with a significant impact on NRAS protein function. Of note, 16 variants were found to be disease-causing and deleterious by all six tools, and the percentage of each SNP type is shown in Supplementary Figure S3. Among these 16 variants, one SNP (rs121434595) was found to have two mutant alleles, and both alleles could result in amino acid substitution (G13C, G13R). Table 1 shows the prediction and scores generated by the six tools for these 16 SNPs.

**TABLE 1:** Prediction and scores of deleterious missense SNPs by six *in silico* tools.

	SNP Id	AA change	SIFT		PolyPhen-2		PROVEAN		SNP&GO		PHD-SNP		SNAP2	
			Prediction	Score	Prediction	Score	Prediction	Score	Prediction	Ri score	Prediction	Ri score	Prediction	Score
1	rs1658971260	R164C	deleterious	0.00	Probably damaging	0.997	Deleterious	−4.975	Disease	3	Disease	8	effect	43
2	rs757968407	V152F	deleterious	0.00	Probably damaging	0.991	Deleterious	−4.484	Disease	2	Disease	8	effect	30
3	rs1163400692	S145L	deleterious	0.00	Probably damaging	0.994	Deleterious	−5.529	Disease	1	Disease	7	effect	42
4	rs754428086	D119G	deleterious	0.00	Probably damaging	0.994	Deleterious	−6.472	Disease	2	Disease	7	effect	45
5	rs1365635887	R68G	deleterious	0.00	Probably damaging	0.979	Deleterious	−6.912	Disease	2	Disease	4	effect	62
6	rs752508313	Y64D	deleterious	0.00	Probably damaging	0.999	Deleterious	−8.841	Disease	7	Disease	8	effect	73
7	rs267606920	G60E	deleterious	0.00	Probably damaging	1	Deleterious	−7.492	Disease	6	Disease	7	effect	36
8	rs1557982817	G60R	deleterious	0.00	Probably damaging	1	Deleterious	−7.492	Disease	5	Disease	8	effect	37
9	rs1465850103	D57N	deleterious	0.00	Probably damaging	0.975	Deleterious	−4.517	Disease	2	Disease	8	effect	64
10	rs1246727247	I55R	deleterious	0.00	Probably damaging	0.996	Deleterious	−6.527	Disease	2	Disease	8	effect	33
11	rs139287106	S39F	deleterious	0.00	Probably damaging	0.997	Deleterious	−5.427	Disease	0	Disease	3	effect	30
12	rs397514553	P34R	deleterious	0.00	Probably damaging	1	Deleterious	−7.698	Disease	5	Disease	5	effect	19
13	rs121913248	A18P	deleterious	0.00	Probably damaging	0.992	Deleterious	−4.316	Disease	7	Disease	10	effect	15
14	rs1308441238	V14G	deleterious	0.00	Probably damaging	0.999	Deleterious	−5.856	Disease	2	Disease	9	effect	60
15	rs121434596	G13V	deleterious	0.00	Probably damaging	0.975	Deleterious	−7.649	Disease	8	Disease	9	effect	65
16	rs121434595	G13C	deleterious	0.00	Probably damaging	0.994	Deleterious	−7.719	Disease	7	Disease	9	effect	59
		G13R	deleterious	0.00	Probably damaging	0.982	Deleterious	−6.709	Disease	7	Disease	9	effect	58

### Prediction of Single Nucleotide Polymorphism Impact on NRAS Protein Stability

The impacts of SNPs on NRAS protein stability were analyzed using the I-Mutant 2.0 server and the Mu-Pro tool. The I-Mutant 2.0 server found that 12 SNPs had a decreasing effect on NRAS protein stability, while the Mu-Pro tool showed a decreasing effect with all SNPs except rs1163400692 SNP. The predicted impacts and values are shown in Table 2 for the 16 SNPs.

**TABLE 2:** Effect of missense variants on NRAS protein stability.

SNP Id	AA change	I-mutant 2 prediction	Reliability index (RI)	DDG value (kcal/mol)	MUpro	
					Prediction	Delta delta G
rs1658971260	R164C	Decrease	5	−1.19	Decrease	−0.17
rs757968407	V152F	Decrease	9	−2.13	Decrease	−0.85
rs1163400692	S145L	Increase	3	0.24	Increase	0.09
rs754428086	D119G	Decrease	7	−0.53	Decrease	−1.43
rs1365635887	R68G	Decrease	6	−0.91	Decrease	−1.92
rs752508313	Y64D	Decrease	4	−0.99	Decrease	−0.90
rs267606920	G60E	Decrease	1	−0.89	Decrease	−0.46
rs1557982817	G60R	Decrease	7	−1.36	Decrease	−0.63
rs1465850103	D57N	Increase	1	0.18	Decrease	−0.65
rs1246727247	I55R	Decrease	7	−2.31	Decrease	−2.01
rs139287106	S39F	Increase	3	0.06	Decrease	−0.24
rs397514553	P34R	Decrease	6	−0.6	Decrease	−0.98
rs121913248	A18P	Increase	1	−1.53	Decrease	−1.32
rs1308441238	V14G	Decrease	10	−4.96	Decrease	−2.02
rs121434596	G13V	Increase	2	−0.02	Decrease	−0.25
rs121434595	G13C	Decrease	5	−1.18	Decrease	−0.32
	G13R	Decrease	6	−1.27	Decrease	−0.15

### Identification of Single Nucleotide Polymorphism Location on NRAS Domains

The InterPro tool analyzed NRAS protein functionally and revealed the presence of an important domain: a small GTP-binding protein domain (InterPro entry: IPR005225). The positions of the 16 deleterious variants were analyzed, and all SNPs were found to be located in this domain, except for rs1658971260, as shown in Table 3.

**TABLE 3:** Locations of *NRAS* variants on protein domains, phylogenetic conservation analysis, and secondary structure prediction.

SNP Id	AA change	Location on protein	ConSurf conservation score	Functional/structural	Buried/exposed	Secondary structure
rs1658971260	R164C	—	5/intermediately conserved	—	exposed	Alpha helix
rs757968407	V152F	Small GTP-binding protein domain	9/highly conserved	structural	buried	Alpha helix
rs1163400692	S145L	Small GTP-binding protein domain	9/highly conserved	functional	exposed	Alpha helix
rs754428086	D119G	Small GTP-binding protein domain	9/highly conserved	functional	exposed	Random coil
rs1365635887	R68G	Small GTP-binding protein domain	7/highly conserved	—	exposed	Alpha helix
rs752508313	Y64D	Small GTP-binding protein domain	8/highly conserved	—	buried	Alpha helix
rs267606920	G60E	Small GTP-binding protein domain	9/highly conserved	functional	exposed	Random coil
rs1557982817	G60R	Small GTP-binding protein domain	9/highly conserved	functional	exposed	Random coil
rs1465850103	D57N	Small GTP-binding protein domain	9/highly conserved	functional	exposed	Alpha helix
rs1246727247	I55R	Small GTP-binding protein domain	8/highly conserved	—	buried	Alpha helix
rs139287106	S39F	Small GTP-binding protein domain	7/highly conserved	—	exposed	Alpha helix
rs397514553	P34R	Small GTP-binding protein domain	7/highly conserved	—	exposed	Random coil
rs121913248	A18P	Small GTP-binding protein domain	8/highly conserved	—	buried	Alpha helix
rs1308441238	V14G	Small GTP-binding protein domain	9/highly conserved	functional	exposed	Random coil
rs121434596	G13V	Small GTP-binding protein domain	7/highly conserved	—	exposed	Random coil
rs121434595	G13C	Small GTP-binding protein domain	7/highly conserved	—	exposed	Random coil
	G13R	Small GTP-binding protein domain	7/highly conserved	—	exposed	Random coil

### Identification of Phylogenetically Conserved Residues in NRAS Protein

The evolutionary conservation of NRAS protein amino acids was analyzed as displayed in Supplementary Figure S4. All SNPs were located in highly conserved positions, except rs1658971260 SNP, which was located in an intermediately conserved position, as demonstrated in Table 3. In addition, six SNPs were located on functional and exposed residues (rs1163400692, rs754428086, rs267606920, rs1557982817, rs1465850103, rs1308441238), while one SNP was located on a structural and buried residue (rs757968407).

### Analysis of Secondary Structures of NRAS Protein

The predicted secondary structures of NRAS were analyzed via the SOPMA tool as displayed in Supplementary Figure S5. SOPMA revealed that 86 residues were associated with alpha helix (45.5%), 55 with random coil (29.1%), 35 with extended strand (18.52%), and 13 with beta-turn (6.88%). Furthermore, analysis of the SNPs’ alignment in the secondary structures was performed as displayed in Supplementary Figure S6; nine deleterious SNPs existed in the alpha helix secondary structure, and five deleterious SNPs existed in the random coil secondary structure. Meanwhile, no deleterious SNPs were found in the beta-turn or the extended strand. The detailed alignment of the SNPs is shown in Table 3.

### Structural Analysis of Predicted NRAS Mutated Models

To predict the tertiary structure of the models with 17 different mutations, SWISS-MODEL presented the wild-type NRAS 3D model with PDB ID of 5UHV as the best choice for homology modeling with 99.39% identity with all the mutated submitted sequences. Validation of these models was performed by detecting the ProSA Z score and the percentage of residues in the favored region for each molecule (Table 4 and Supplementary Figure S7), where the corresponding scores for all predicted models confirmed the high quality of the predicted models. Regarding the structural similarity between the native and predicted models, the high values of TM (close to one for all predicted models) and the small scores of RMSD (close to zero for all predicted models) demonstrated the high similarity between the native model and the designed ones (Table 4). This was reasonable as SWISS-MODEL in the first place selected a native structure with high similarity with the submitted mutated sequences to build the required models, and this small variation came from the substitution of one amino acid that represents the SNP.

**TABLE 4:** Scores of validation, structural similarity, and binding affinity of the predicted mutant models.

No.	SNP	Ramachan assessment (favored)	ProSA Z score (%)	Tm-score	RMSD	GTP-binding score (kcal/mol)
1	G13R	96.93	−7.38	0.99384	0.06	−12.3
2	G13C	96.93	−7.36	0.99383	0.06	−12
3	G13V	96.32	−7.38	0.99383	0.06	−12.2
4	V14G	96.93	−7.30	0.99384	0.06	−11.7
5	A18P	96.32	−7.51	0.99365	0.09	−11.6
6	P34R	96.32	−7.10	0.99383	0.06	−12
7	S39F	96.93	−7.29	0.99384	0.06	−11.8
8	I55R	96.32	−7.12	0.99365	0.09	−11.7
9	D57N	97.55	−7.52	0.99384	0.06	−11.6
10	G60R	96.32	−7.32	0.99302	0.15	−11.5
11	G60E	96.32	−7.19	0.99344	0.11	−10.6
12	Y64D	96.93	−7.38	0.99383	0.06	−11.6
13	R68G	96.93	−7.42	0.99383	0.06	−11.9
14	D119G	96.93	−7.35	0.99383	0.06	−11.8
15	S145L	97.55	−7.40	0.99374	0.07	−11.1
16	V152F	96.32	−7.63	0.99348	0.11	−12
17	R164C	96.93	−7.32	0.99383	0.06	−11.8

### Molecular Docking Analysis

The current docking study was performed to demonstrate the difference in the binding affinity between native and mutated models with the GTP ligand. The binding score between the native NRAS and GTP was −11.3 kcal/mol, and for the mutant predicted structures, the respective binding score is shown in Table 4. Analysis of the scores showed that all models, except models #11 and #15, exhibited a higher affinity to GTP than the native model, where models #1, #2, #3, #6, and #16 were at the top of the list regarding their binding affinity to GTP.

**FIGURE 1:**
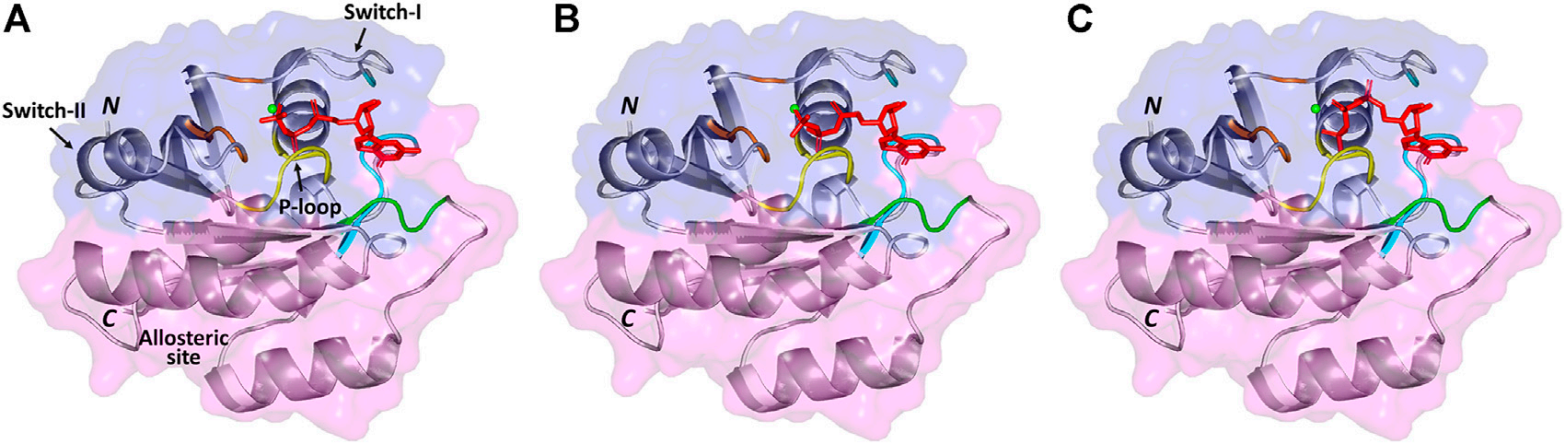
3D structure of GTP (red color sticks) docked in NRAS target receptor (cartoon and surface) with Mg^2+^ ion (green sphere). **(A)** Native model, **(B)** mutant model with the highest binding affinity (G13R SNP), and **(C)** mutant model with the lowest binding affinity (G60E SNP). NRAS cartoon/surface is colored differently according to its constitutive halves: effector lobe (blue) and allosteric lobe (magenta). P-loop (residue range: 10–17), switch-I (residue range: 30–40), and switch-II (residue range: 60–76) for the GTP-binding site. Conserved GTPase motifs reported essential for ligand recognition/binding and Mg^2+^ coordination are colored differently; GxxxxGK (yellow), DxxG and Thr35 (brown), NKxD (green), as well as ExSAK and Phe28 (cyan). Letters *N* and *C* denote the amine- and carboxy-protein terminals, respectively.

The 3D complexes of the native model, model #1 (the one with the highest binding affinity), and model #11 (the one with the lowest binding affinity) with GTP are visualized by PyMOL, shown in Figure 1, while a presentation of reacting residues of the same models is shown as a 2D chart by LIGPLOT (Supplementary Figure S8). The general topology of the predicted models illustrated the distinctive two lobes (effector lobe: 1–86 residues and allosteric lobe: 87–166 residues) of the GTPase catalytic domain. The active site for GTP-binding is formed via the P-loop, Switch-I, and switch-II with residue ranges of 10–17, 30–40, and 60–76 amino acids, respectively (Johnson et al., 2017). The GTPase-conserved motifs at the effector lobe, P-loop GxxxxGK (residues 10–16), and DxxG/Thr35 (residues 57–60) were shown to support the GTP-α-β-phosphate groups and Mg2+ coordination (Araki et al., 2011). On the other half, the allosteric lobe showed the other GTPase-conserved motifs including NKxD (residues 116–119) responsible for guanine nucleotide specificity, whereas, ExSAK (residues 143–147) together with Phe28 being responsible for stabilized nucleotide binding, respectively (Parker and Mattos, 2015).

### Molecular Dynamics Investigation

**FIGURE 2:**
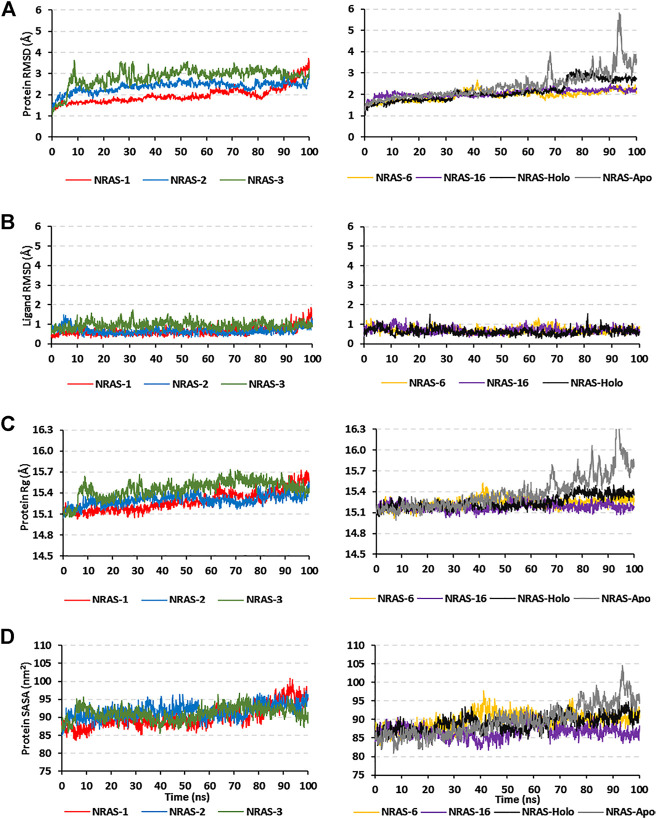
Trajectory analysis for the simulated GTPase NRAS models. Monitored alpha-carbon trajectories of **(A)** protein’s RMSD, **(B)** ligand’s RMSD, **(C)** protein’s Rg, and **(D)** protein’s SASA were plotted against the entire molecular dynamics simulation timelines (100 ns).

Simulated proteins of the GTPase NRAS models showed steady equilibrated RMSD trajectories, respective to initial proteins’ alpha-carbons, across the 100-ns runs (Figure 2A). The steadiest alpha-carbon RMSDs were depicted for simulated model numbers 2, 6, and 16 as compared to other models, with average tone values of 1.98 ± 0.39 Å, 1.95 ± 0.23 Å, and 2.06 ± 0.17 Å, respectively. Notably, all simulated NRAS models were of much-equilibrated RMSD tones as compared to the native NRAS both at its respective holo (GTP bounded: 2.13 ± 0.45 Å) and apo (unliganded: 2.41 ± 0.65 Å) states. The end molecular dynamics timeframe (60–100 ns) showed high RMSD fluctuations for the apo/unliganded NRAS model, reaching up to ∼5.70 Å. On the other hand, the holo native NRAS showed a slight increase in the protein RMSD following the 75-ns timeframe and till the end of the simulation run.

Monitoring the RMSD trajectories of the GTP ligand, in relation to its initial/reference position, showed steady tones for the simulated models (Figure 2B). The RMSDs of the ligand were much lower than those of their respective bounded proteins, the thing that conferred stable GTP-pocket accommodation across the whole 100-ns simulation runs. The simulated models were further investigated by monitoring the protein’s Rg and SASA trajectories (Figures 2C, D). Low average Rg—SASA values, 15.30 ± 0.08 Å//91.64 ± 1.96 nm^2^, 15.24 ± 0.07 Å//89.64 ± 2.25 nm^2^, and 15.19 ± 0.05 Å//86.69 ± 1.74 nm^2^, were assigned for the simulated model numbers 2, 6, and 16, respectively. Again, the native holo NRAS protein showed a slight increase in its Rg and SASA tones, which then proceeded constantly till the end of the simulation runs. Similar to the protein’s RMSDs, the monitored SASA trajectories were of the highest values for the simulated apo native NRAS protein as compared to other simulated congerants.

**FIGURE 3:**
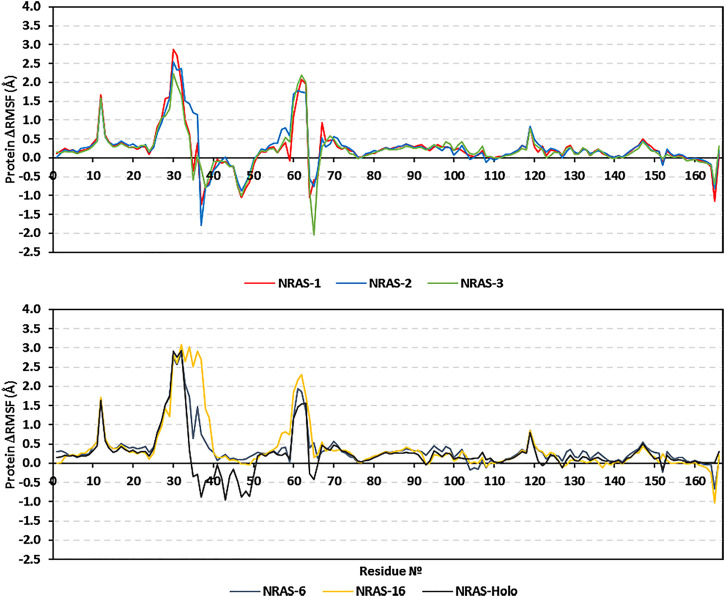
Relative ΔRMSF analysis for the simulated GTP-bounded NRAS proteins along the whole molecular dynamics simulations. The ΔRMSF values, in reference to protein backbone Cα-atoms, are represented in terms of constituting residue sequence numbers.

Dissecting the stability profile of the simulated GTPase NRAS proteins into their respective residue-oriented level was performed by monitoring the alpha-carbon RMSF trajectories. Difference RMSF values for a particular GTP-NRAS model in relation to the native apoprotein (ΔRMSF = RMSF_Apo_—RMSF_Holo_) was of better representation for the residue-wise protein stability and fluctuation. Residues depicting ΔRMSF values ≥ 0.3 cut-offs were considered significantly rigid, showing negligible mobility (Albuquerque et al., 2020). Notably, the simulated proteins showed typical thermodynamic behavior, where terminal residues were of high negative ΔRMSF values as compared to most of the core amino acids (Figure 3). On the other hand, certain effector lobe residue ranges depicted high immobility profiles being confined with the GTPase-binding site. These top-immobile residue ranges were the P-loop GxxxGK motif (residues 10–16), switch-I, and vicinal amino acids (residues 26–39), as well as the switch-II range (residues 60–70) with the reported Mg^+2^-coordinating DXXG motif (Table 5).

**TABLE 5:** Monitored ΔRMSF^a^ [Å] for GTPase NRAS complexes along entire molecular dynamics runs.

Canonical domains composing GTPase-binding/catalytic site	Residues	Model #1	Model #2	Model #3	Model #6	Model #16	Native holo
*N*-terminal P-loop GxxxGK motif	**Gly10**	0.38	0.35	0.32	0.42	0.42	0.32
	**Ala11**	0.52	0.45	0.45	0.56	0.56	0.45
	**Gly12**	1.67	1.58	1.59	1.68	1.72	1.64
	**Gly/x13**	0.60	0.55	0.56	0.62	0.66	0.60
	**Val14**	0.43	0.41	0.40	0.45	0.46	0.40
	**Gly15**	0.34	0.34	0.30	0.37	0.38	0.29
	**Lys16**	0.35	0.36	0.30	0.40	0.37	0.33
	**Ser17**	0.41	0.44	0.38	0.51	0.48	0.43
Switch-I vicinal residues	**Gln25**	0.35	**0.27**	0.34	0.42	0.27	0.38
	**Asn26**	0.81	0.66	0.73	0.76	0.69	0.80
	**His27**	1.04	0.93	1.06	0.98	0.94	1.09
	**Phe28**	1.57	1.23	1.11	1.52	1.43	1.52
	**Val29**	1.61	1.60	1.31	1.77	1.22	1.73
Switch-I	**Asp30**	2.88	2.53	2.23	2.73	2.83	2.90
	**Glu31**	2.71	2.33	1.95	2.58	2.61	2.76
	**Tyr32**	2.00	2.38	1.65	2.87	3.07	2.94
	**Asp33**	1.01	1.51	0.91	2.08	2.64	1.83
	**Pro/Arg34**	0.64	1.44	0.59	1.74	3.02	0.33
	**Thr35**	**−0.35**	1.21	**−0.58**	0.64	2.52	**−0.35**
	**Ile36**	0.38	1.14	**0.06**	1.47	2.90	**−0.28**
	**Glu37**	**−1.24**	**−1.79**	**−0.28**	0.76	2.71	**−0.88**
	**Asp38**	**−0.76**	**−0.78**	**−0.80**	0.55	1.41	**−0.46**
	**Ser39**	**−0.65**	**−0.72**	**−0.46**	0.38	1.18	**−0.41**
	**Tyr40**	**−0.29**	**−0.34**	**−0.10**	**0.28**	**0.27**	**−0.40**
DxxG motif	**Asp57**	**0.29**	0.75	0.34	0.40	0.78	**0.21**
	**Thr58**	0.40	0.80	0.55	0.41	0.81	**0.26**
	**Ala59**	**−0.07**	0.57	0.42	**0.02**	0.74	**0.12**
	**Gly60**	1.09	1.69	1.49	1.20	1.84	1.15
Switch-II	**Gln61**	1.71	1.79	1.95	1.95	2.17	1.45
	**Glu62**	2.08	1.74	2.18	1.87	2.30	1.53
	**Glu63**	1.97	1.72	2.00	1.34	1.74	1.56
	**Tyr-64**	**−1.05**	**−0.54**	**−0.88**	0.40	1.18	**−0.27**
	**Ser65**	**−0.59**	**−0.76**	**−2.03**	0.54	**0.15**	**−0.43**
	**Ala66**	**−0.54**	**−0.33**	**−0.50**	**0.14**	**0.23**	**−0.04**
	**Met67**	0.94	0.52	**0.28**	0.30	0.55	0.48
	**Arg68**	0.45	**0.29**	0.43	0.34	0.31	0.40
	**Asp69**	0.46	0.36	0.58	0.43	0.35	0.33
	**Gln70**	0.46	0.56	0.50	0.57	0.33	0.47
	**Tyr71**	**0.28**	0.53	0.34	0.48	0.35	0.43
	**Met72**	**0.23**	0.33	**0.25**	0.31	0.34	0.32
	**Arg73**	**0.29**	0.31	**0.27**	**0.26**	0.33	0.30
	**Thr74**	**0.19**	0.24	**0.12**	**0.17**	**0.28**	**0.23**
	**Gly75**	**0.17**	**0.17**	**0.09**	**0.15**	**0.18**	**0.18**
	**Glu76**	**0.02**	**0.02**	**−0.01**	**0.02**	**0.03**	**0.05**
NKxD motif	**Asn116**	**0.22**	**0.22**	**0.18**	**0.26**	**0.23**	**0.22**
	**Lys17**	0.32	0.32	**0.26**	0.36	0.34	0.31
	**Cys118**	**0.27**	**0.28**	**0.22**	0.28	**0.29**	**0.27**
	**Asp119**	0.82	0.84	0.78	0.86	0.86	0.80
*C*-terminal ExSAK motif	**Glu143**	**0.12**	**0.12**	**0.05**	**0.16**	**0.09**	**0.12**
	**Thr144**	**0.18**	**0.19**	**0.12**	**0.25**	**0.15**	**0.15**
	**Ser145**	**0.28**	**0.27**	**0.19**	0.32	**0.26**	**0.26**
	**Ala146**	0.34	0.32	**0.25**	0.38	**0.26**	0.33
	**Lys147**	0.51	0.44	0.43	0.55	0.44	0.52
SNP in model #16	**Val/Phe152**	**−0.16**	**−0.20**	**−0.03**	**−0.24**	**0.24**	**−0.17**

a ΔRMSF ≤ 0.30 Å cut-off are in bold red numbers inferring residues showing significant mobility/flexibility.

Moving toward the allosteric lobe residues, the two motifs responsible for guanine nucleotide specificity and stabilized nucleotide binding (NKxD and ExSAK GTPase motifs) depicted a significant immobility profile with ΔRMSF above the 0.30 Å cut-off value. Notably, stability residue-wise ranges were more prominent for the effector lobe rather than those at the allosteric half of the GTPase catalytic domain. Concerning the comparative NRAS flexibility profiles, model #3, along with the native holo protein, showed the highest mobility tones with more negative and less positive ΔRMSF values. It is interesting that SNP NRAS models #1, #2, and #3 depicted consistent mobility and flexibility profiles for the 37–50 residue range, showing almost all its respective residues with negative ΔRMSF values. Moreover, selected switch-II residues (Tyr64, Ser65, Ala66) exhibited high ΔRMSF values for the same three Gly13 SNP models. It is worth noting that the native holo protein depicted negative ΔRMSF values for the same previous switch-I and -II residue ranges yet at less prominent negative numbers. On different bases, the three Gly13 SNP residues, G13R, G13C, and G13V, in models #1, #2, and #3, respectively, showed a slightly higher immobility profile (ΔRMSF ∼0.60 Å) than their native Gly13 in holo NRAS protein. The P34R SNP residue in model #6 showed a more preferential stability profile than its native Pro34 congerant. Likewise, the V152F SNP in model #16 was more rigid, reaching a positive ΔRMSF value (0.24 Å), as compared to its negative-valued native residue (Val152).

**FIGURE 4:**
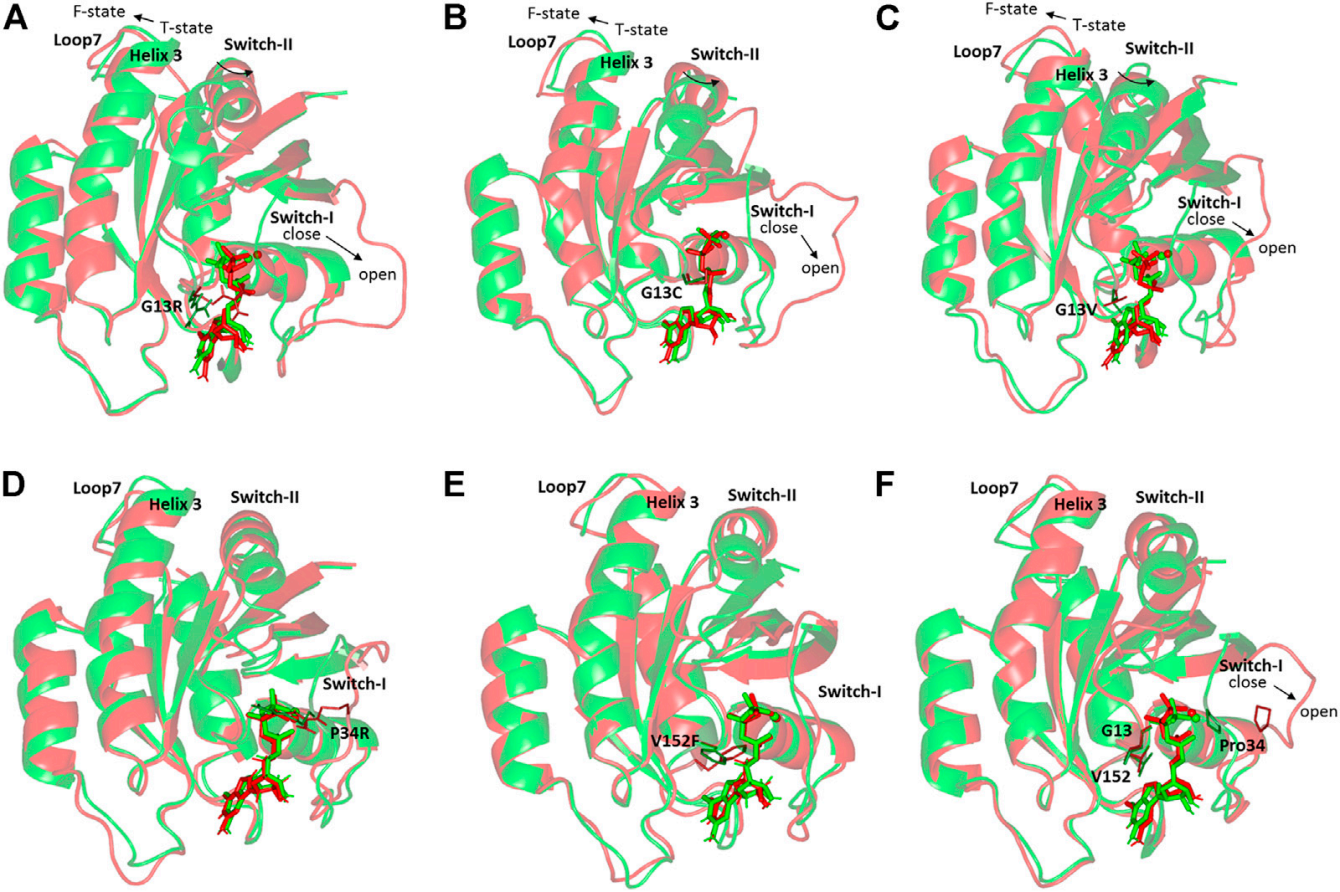
Conformational analysis of simulated GTPase NRAS-GTP complexes at the start and final molecular dynamics timeframes. Superimposed 0 and 100-ns shots of the simulated NRAS complexes; **(A)** model #1, **(B)** model #2, **(C)** model #3, **(D)** model #6, **(E)** model #16, **(F)** native holo protein. Complexes are shown in green and red cartoons respective to the initial and last extracted frames. Ligands (sticks) and SNP residues (lines) are presented in colors corresponding to extracted frames.

Conformational analysis for the simulated NRAS was performed to grasp the main secondary structure changes and SNP residue conformational shift throughout the molecular dynamics simulations. Trajectories at the start and end of the simulation runs were extracted, minimized at 0.0001, and finally overlayed. As expected, the docked GTP molecule was attained within the NRAS-binding site at the end of molecular dynamics simulation runs (Figure 4). Most SNP residues showed limited orientation/conformational changes at the end of the simulation runs. The latter was obvious for G13C, G13V, and V152F in models #2, #3, and #16, respectively. Nevertheless, the G13R SNP residue in model #1 exhibited the highest conformational drift (alpha-carbon RMSD = 1.034 Å) as compared to its G13 SNP congerants (alpha-carbon RMSD ∼ 0.800 Å). The G13R residue showed closer proximity to the GTP phosphate groups as compared to its start frame, favoring phosphate group stability and extended hydrogen bonding. Concerning the P34R SNP residue in model #6, a moderate conformational shift was depicted for this residue with a depicted alpha-carbon RMSD of 0.941 Å.

It is worth mentioning that significant secondary structure conformational alterations were also depicted and such observations were differential across each simulated model. Regarding the G13x SNP model, a significant shift of the switch-I loop of the simulated NRAS proteins at the end of the molecular simulation runs. A switch from the closed state-1 to an open state-2 was depicted with models #1–3, being more obvious for model #1. This switch-I drift was accompanied by an outward movement for the α-helix 3 and loop 7 away from the switch-II domain. The latter drift permitted the switch-II to attain the catalytically active R-state conformation rather than the catalytically incompetent T-state at the start of simulation runs. The above-described switch-I and switch-II movements were less prominent with SNP models #6 and #16, where minimal or insignificant conformational changes were depicted. The native holo NRAS complex exhibited moderate switch-I drift toward the open state-1, yet without significant outward movement of its α-helix 3/loop 7 nor its switch-II domain.

Estimating the free binding affinity of GTP toward the constructed NRAS models as well as the native holo state was performed using the trajectory-oriented MM-PBSA calculations. Using the single trajectory approach and SASA-only model (ΔG Total = ΔG Apolar + ΔG Polar + ΔG Molecular Mechanics), the total binding energies for GTP in all SNP NRAS models were superior to that of the native holo protein (Table 6). This SNP-favored binding affinity is consistent with the results of the preliminary molecular docking investigation. The obtained free binding energies were dissected into their contributing energy terms, showing a general trend in several GTPase NRSA complexes. Dominant energy contributions of the van der Waals interactions (ΔG Van der Waals) over that of the Coulomb’s electrostatic potentials (ΔG Electrostatics) were depicted for SNP models #2, #3, and #16 as well as the native holo complex. The latter van der Waal energy preferentiality was most obvious for SNP model #6. On the other hand, both SNP models #1 and #6 depicted almost two-fold higher ΔG Electrostatics contribution over the hydrophobic potentials, the thing that was also remarkably associated with high polar solvation energies (ΔG Solvation; Polar). Moreover, the non-polar solvation energy terms were almost consistent for all simulated NRAS complexes, being around 19.0 kJ/mol.

**TABLE 6:** The MM-PBSA-calculated total binding-free energy and its constituting energy terms.

Energy (kJ/mol ± SD)	GTP-NRAS complexes					
	Model #1	Model #2	Model #3	Model #6	Model #16	Native holo
Δ*G*_van der Waals_	**−**180.4 ± 18.9	**−**189.9 ± 13.8	**−**178.1 ± 19.4	**−**176.6 ± 25.1	**−**228.2 ± 25.2	**−**198.0 ± 28.0
Δ*G*_Electrostatics_	**−**238.1 ± 35.1	**−**89.0 ± 51.3	**−**44.6 ± 41.9	**−**291.5 ± 41.3	**−**64.2 ± 69.1	**−**39.3 ± 69.8
Δ*G*_Solvation;_ _Polar_	316.7 ± 14.2	131.5 ± 29.0	113.9 ± 28.4	336.5 ± 39.1	174.5 ± 29.2	163.2 ± 12.3
Δ*G*_Solvation; non-polar; SASA_	**−**20.2 ± 0.6	**−**19.5 ± 0.4	**−**18.7 ± 1.6	**−**19.1 ± 0.4	**−**18.6 ± 0.2	**−**19.4 ± 0.2
Δ*G*_Total binding_	**−**122.0 ± 38.0	**−**166.9 ± 48.4	**−**127.5 ± 49.5	**−**150.7 ± 58.8	**−**136.5 ± 22.8	**−**93.5 ± 39.9

**FIGURE 5:**
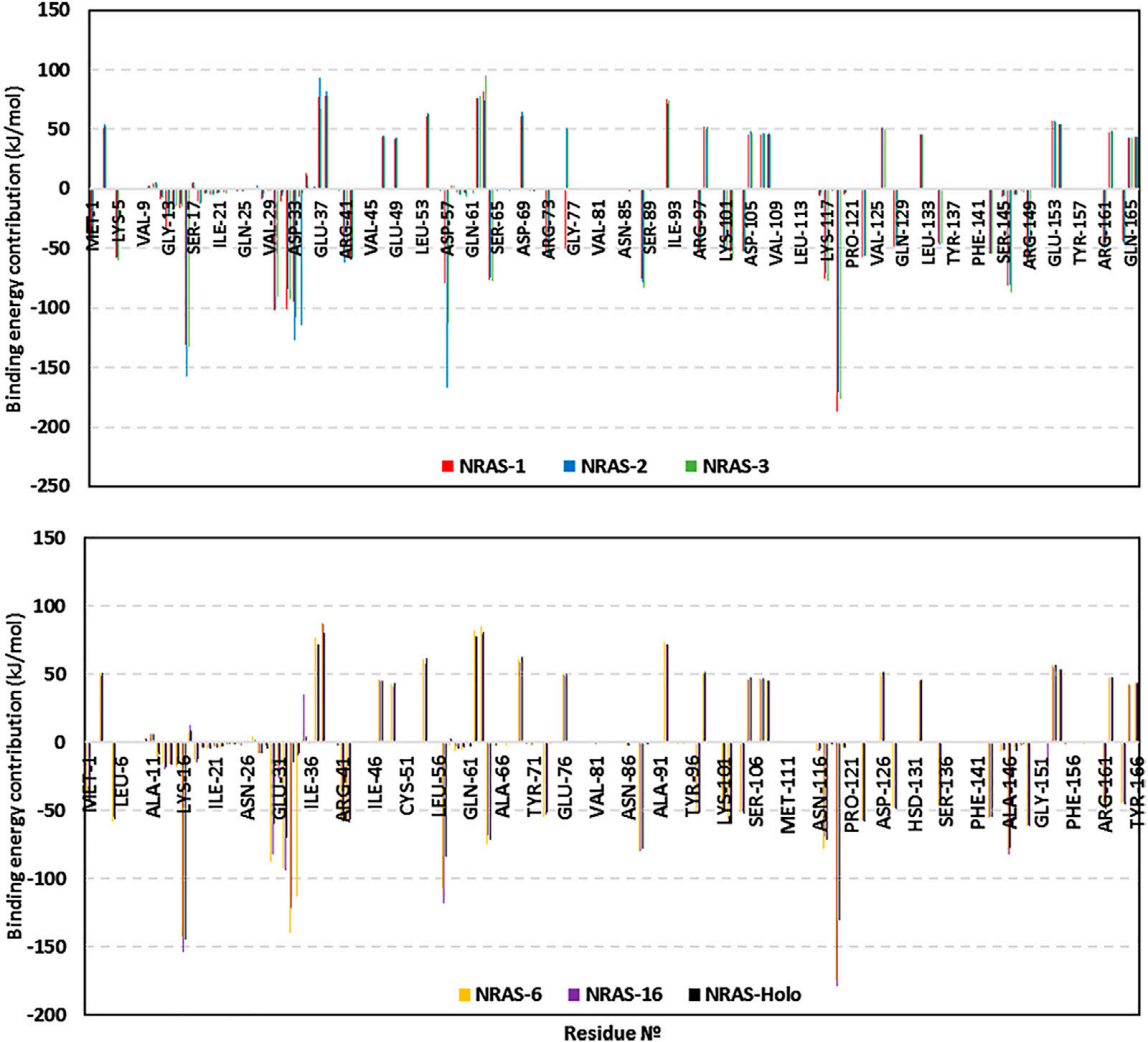
Residue-oriented free binding energies for the simulated GTP-NRAS systems.

The obtained total binding-free energies were further decomposed down to the residue-wise binding energy contributions (Figure 5) to identify the key binding residues. The highest negative energy contribution values were assigned to residues of the above-described motifs being confined with GTP-binding and recognition. The P-loop Lys16 of the GxxxxGK motif and Asp119 of the NKxD motif depicted the highest residue-wise energy contributions across all simulated NRAS complexes, with values of −131.2 up to −157.4 kJ/mol and −130.9 up to −187.5 kJ/mol, respectively. Other GTP active site residues showed significant energy term contributions, including Val14, Ala18, Phe28, Asp30, Tyr32, Asp57, Tyr64, Lys117, and/or Ala146.

Most of the latter top-energy-contributing polar residues showed high hydrogen bond % frequencies with GTP throughout the entire simulation runs using the VMD hydrogen–bond tools. **SNP model #1:** Arg13-side (30.64%), Lys16-side (93.41%), Ala18-main (44.31%), Asp30-main (30.34%), Asp119-side (123.05%); **SNP model #2:** Cys13-side/main (22.45%), Lys16-side (69.06%), Ala18-main (14.22%), Asp30-side (50.82%), Asp119-side (118.66%); **SNP model #3:** Lys16-side/main (107.29%), Ala18-main (52.10%), Asp119-side (123.45%); **SNP model #6:** Gly13-main (30.84%), Lys16-side/main (131.62%), Ala18-main (34.73%), Asp30-main (32.63%), Tyr32-side (53.29%), Arg34-side (38.82%), Asp119-side (123.25%); **SNP model #16:** Lys16-side/main (132.23%), Tyr32-side (63.97%), Asp119-side (115.87%); and **native holo NRAS:** Lys16-side (105.39%), Ala18-main (41.72%), Asp30-main (35.53%), Asp119-side (120.86%).

On the contrary, other hydrophilic pocket-lining residues, including Glu37, Arg38, Glu62, Glu63, and/or Asp69, exhibited unfavored positive-valued energies. The latter binding repellent residues were of the highest values for SNP models #1 and #6. Concerning the SNP residues, G13R showed a higher negative energy contribution in model #1 as compared to its SNP or native congerants. Likewise, model #6 showed significant binding energy for its SNP P34R, with a value reaching up to −113.0 kJ/mol. The SNP V152F residue showed relevant energy contribution in model #16, whereas the native congerant illustrated either poor energy contributions (∼−0.45 kJ/mol) or even positive-value energies (∼0.14 kJ/mol).

### Normal Mode Analyses

Collective functional flexibility/mobility of modeled SNP and native holo NRAS proteins was assessed via the iMODS server and represented in Supplementary Figure S9. In particular, the residue range 33–55, corresponding to the switch-I domain and vicinal residues, depicted the highest deformability across all NRAS proteins (Supplementary Figure S9A). This inherited flexibility pattern was also recapitulated through the estimated highest B-factor scores for these residue regions (Supplementary Figure S9B). The B-factor analytical parameter can quantify the atomic displacement amplitudes around a conformational equilibrium (López-Blanco et al., 2014). The estimated eigenvalues (1) of SNP models #1, #2, and #3 complexes were the highest (∼8.8e^−04^) as compared to those of other SNP models as well as the native NRAS protein (Supplementary Figure S9C). In addition, the depicted inverse eigenvalue-variance relationship related to each normal mode predicted a significantly higher intrinsic mobility Gly13 SNP models across the collective function motions (Supplementary Figure S9D).

Regarding the obtained covariance matrices (Supplementary Figure S9E), correlated residue-pair motions (reds) were more assigned to the NRAS proteins as compared to either the uncorrelated (white) or anti-correlated (blue) motions. The depicted correlated residue-pair motions were highly correlated to the switch-I residue ranges as compared to other protein domains. Finally, the elastic network model further described the differential flexibility pattern in Supplementary Figure S9F, where this model illustrated atom-wise pairs linked through springs related to their relative stiffness magnitudes (darker gray strings confer stiffness). Scattered non-continuous dark gray bands, around the normal distribution stiffer string, were assigned for NRAS proteins, particularly the switch-I residues.

### Identification of SNP Impact on NRAS Protein Structure

**FIGURE 6:**
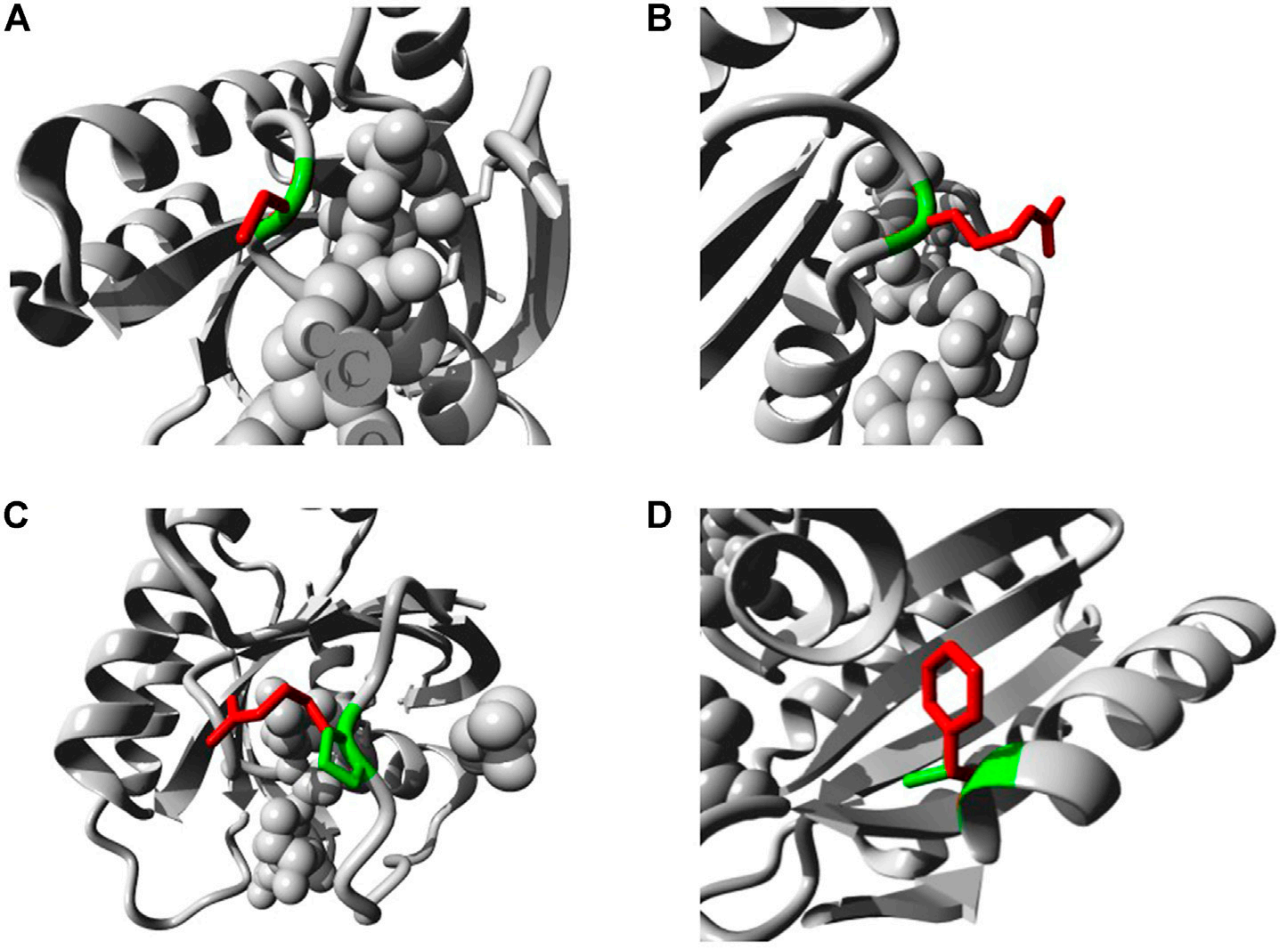
Illustration of the substitution of wild type amino acid (green colored) by the mutant one (red colored) in different NRAS SNPs **(A)** G13C. **(B)** G13R. **(C)** P34R. **(D)** V152F.

The five mutations with the highest binding affinity to GTP were further analyzed for their impacts on NRAS protein structure using the HOPE server. Table 7 explains in detail the different structural impacts in NRAS protein associated with these variants. Structure substitution of wild-type amino acids by mutants is illustrated in Figures 6A–D for G13C, G13R, P34R, and V152F, respectively.

**TABLE 7:** Predicted SNP impacts on NRAS protein structure by HOPE server.

SNP Id	AA change	Amino acid properties	Location/structure	SNP’s impact on the protein
rs757968407	V152F	There is a difference in size as the mutant amino acid is bigger than the wild one. Therefore, the mutant amino acid could not fit as the wild residue is buried in the core	The wild residue is buried in the core. Therefore, the mutant amino acid could not fit due to its bigger size	The mutation affects a domain that is important in binding to other molecules and could disturb its contact with the important domain for the activity of our protein and disturb signal transfer between two domains
rs397514553	P34R	There are differences in charge, size, and hydrophobicity between the mutant residue and the wild one, which could cause repulsions with neighboring residues, disturbance of interactions with protein parts or other molecules, and loss of hydrophobic interactions, respectively	The mutation occurs at a stretch of residues described as a special motif in UniProt, which could suffer from disturbance and loss of function. In addition, the special backbone conformation due to proline rigidity could be disturbed with the mutant residue	The mutation affects a domain that is important in binding to other molecules and could disturb its contact with the important domain for the activity of our protein and disturb signal transfer between two domains
rs121434596	G13V	There is a difference in size as the mutant residue has a bigger size, which could lead to bumps. In addition, the unusual torsion angles by glycine could be lost with disturbance in the local structure, and the local backbone could be forced into incorrect conformation as well	The mutation could lead to a loss of the flexibility of glycine, which may be necessary for the function of the protein	The mutation affects a domain with importance for binding to other molecules. Therefore, this could lead to the disturbance of this function
rs121434595	G13C	There is a difference in size as the mutant amino acid is bigger than the wild one. Therefore, the mutant one could not fit as the wild residue is buried in the core. In addition, the unusual torsion angles by glycine could be lost with disturbance in the local structure, and the local backbone could be forced into incorrect conformation as well	The mutation could lead to a loss of the flexibility of glycine, which may be necessary for the function of the protein	The mutation affects a domain that is important in binding to other molecules and could disturb its contact with the important domain for the activity of our protein and disturb signal transfer between two domains
	G13R	There is a difference in charge between the mutant and wild residues, and the introduced charge in this buried residue could result in protein folding problems. Moreover, there is a difference in size as the mutant amino acid is bigger than the wild one. Therefore, the mutant one could not fit as the wild residue is buried in the core. In addition, the unusual torsion angles by glycine could be lost with disturbance in the local structure, and the local backbone could be forced into incorrect conformation as well	The mutation could lead to a loss of the flexibility of glycine, which may be necessary for the function of the protein	The mutation affects a domain that is important in binding to other molecules and could disturb its contact with the important domain for the activity of our protein and disturb signal transfer between two domains

### Prediction of the Sites of Post-Translational Modification

**FIGURE 7:**
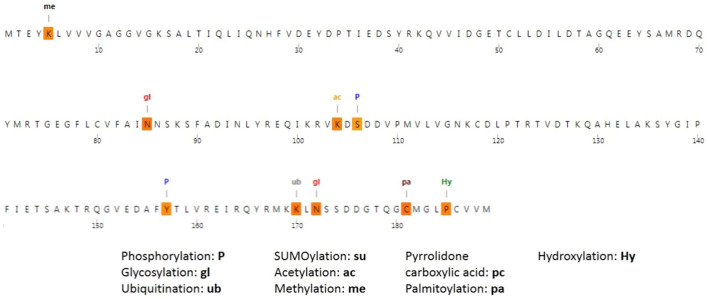
Post-translational modifications of NRAS produced by the MusiteDeep server.

The MusiteDeep server was used to identify the predicted sites of PTM; Figure 7 displays the predicted PTM sites in the NRAS protein. In addition, the analysis predicted the mutant residue C164 to be a palmitoylation site. Moreover, the presence of I55R SNP was found to lead to the appearance of a phosphorylation site at the wild residue of Y64. Furthermore, the presence of D119G SNP was found to lead to the loss of a PTM site at the wild residue of S106.

### Gene–Gene Interaction Analysis

**FIGURE 8:**
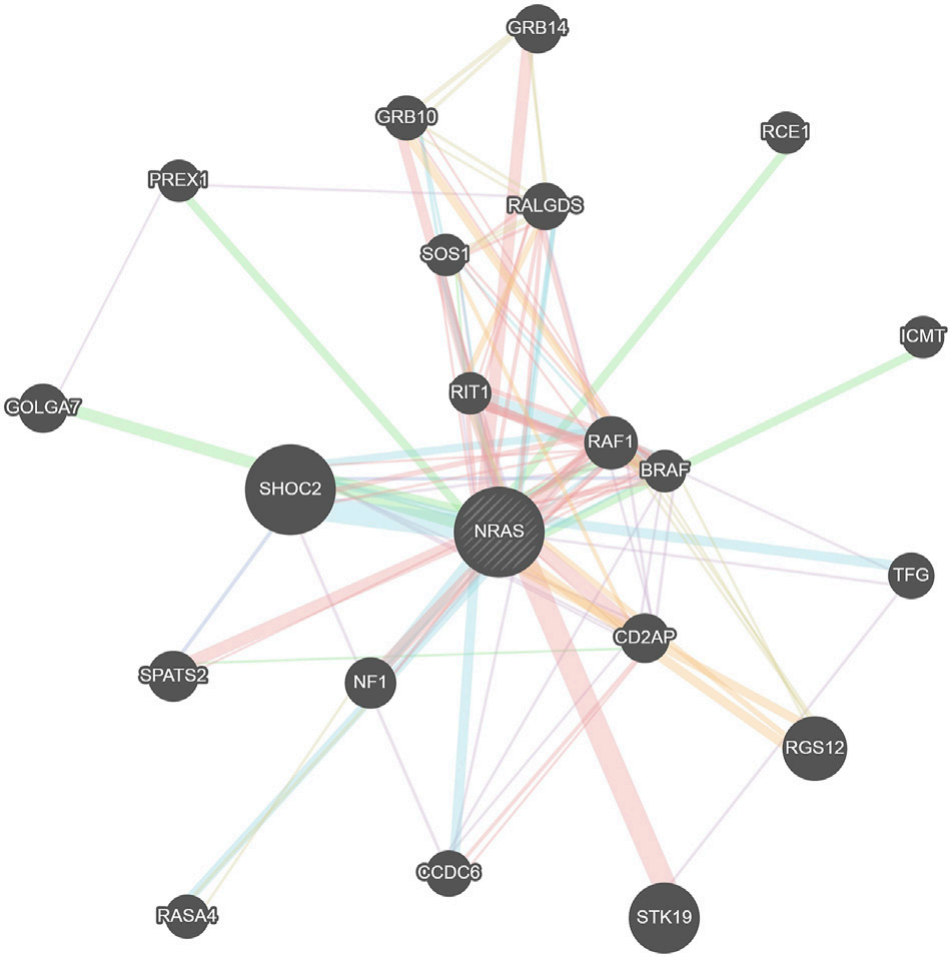
Network of NRAS gene–gene interactions produced by the GeneMANIA tool.

The GeneMANIA tool was used for generating the *NRAS* gene–gene interaction network and identifying the genes that strongly interact with the *NRAS* gene. Figure 8 displays the 20 most strongly connected genes to the *NRAS* gene. Regarding these genes, the *SHOC2* gene (leucine-rich repeat scaffold protein gene) showed the strongest relatedness with a rank of 1, followed by the *STK19* (serine/threonine kinase 19) gene and the *RGS12* (regulator of G protein signaling 12) gene consecutively.

## Discussion

The *NRAS* gene is a well-known oncogene with a major role in carcinogenesis (Eisfeld et al., 2014; Ohashi et al., 2013). *NRAS* gene mutations were found to be associated with aggressive tumor features in many types of tumors, such as melanomas and colorectal cancer (Ellerhorst et al., 2011; Bronte et al., 2015). Moreover, NRAS mutations were proved to be a predictor of poor prognosis, low survival, and reduced response to therapy with different types of tumors as well (Jakob et al., 2012; Bronte et al., 2015). Therefore, it has become necessary to perform a comprehensive *in silico* analysis of the *NRAS* gene to identify the most deleterious missense SNPs with the highest risk of affecting protein structure and function.

By filtering 4,542 SNPs found in the *NRAS* gene, a total of 113 missense SNPs were found, which were further screened using six *in silico* tools with diverse algorithms to augment the efficacy and accuracy of our analysis (SIFT, PolyPhen-2, PROVEAN, SNAP, SNP&GO, and PHD-SNP). In total, 16 SNPs were predicted to be deleterious and disease-causing by all used tools. Due to the critical role of protein stability in protein function and structure (Deller et al., 2016), the impacts of these SNPs on NRAS protein stability were analyzed using the I-Mutant 2.0 server and the Mu-Pro tool. All SNPs were predicted to decrease stability by one server at least, except rs1163400692 SNP.

Furthermore, the InterPro server was used to identify the locations of these SNPs on NRAS protein domains, and the analysis found that all SNPs, except rs1658971260 SNP, were located on the important, small GTP-binding protein domain. This domain is responsible for catalytic activity and contains sites of nucleotide binding and effector interactions (Omerovic et al., 2007; Nussinov et al., 2021). Therefore, mutations in this region are expected to affect the protein function and its binding properties. In addition, phylogenetic analysis was performed using the ConSurf tool, which revealed that all mutations were located on positions with high conservation scores, except the rs1658971260 variant. As residues with functional importance show high conservation scores (Berezin et al., 2004), the existence of variations at these highly conserved positions was predicted to have effects on protein function. Then, the secondary structure alignment analysis showed that all deleterious SNPs were located at the alpha helix and random coil regions, and the highest number of these variants was located at the alpha helix region with nine SNPs.

In addition, we employed a docking analysis to investigate the effect of 17 filtered mutations on the binding affinity with the GTP molecule. A similar approach was employed to study the consequences of two deleterious SNPs on *KRAS*, a member of the Ras family where *NRAS* belongs (Chen et al., 2013). Moreover, several docking studies have been adopted to investigate NRAS as a promising drug target for bis-pyrimidine derivatives (Kumar et al., 2018), 1,3-diazine scaffolds (Kumar et al., 2019), and anticancer agents from *Ocimum basilicum* (Purushothaman et al., 2019); the results of these studies confirmed the possibility of NRAS to act as a target for antitumor agents, which strengthens the need to analyze NRAS structural and ligand affinity alteration as a result of SNPs. The docking analysis in the current study demonstrated that out of 17 mutations, 15 mutations showed a higher binding affinity to GTP compared with the native model, implicating the cell to be in an active proliferation state and increasing the tendency of malignancy. On the other hand, only two SNPs (G60E and S145L) showed a lower affinity.

Analysis of the interacting residues showed that for the native model, 11 residues, namely, Gly13, Val14, Gly15, Lys16, Ser17, Ala18, Gly60, Asn116, Lys117, Asp119, and Ala146, were involved in the reaction with GTP, but for the mutant model G13R, which showed the highest binding affinity for GTP, 12 residues were involved in GTP-binding. These residues were the same as the 11 residues of the native model with the replacement of the Gly13 interacting residue with Arg13 and adding Thr35 as a new interacting residue. The replacement and the new interacting residue increased the binding affinity from −11.3 to −12.3. On the other hand, a comparison between the native and mutant model G60E, which demonstrated the lowest binding affinity for GTP, showed a reduction for interacting residues from 11 to 7 with a concurrent reduction in the binding affinity for GTP from −11.3 to −10.6. Altogether, the molecular docking study revealed that 15 out of the 17 filtered mutations have a high potential to affect the interaction between NRAS and GTP, which would reflect on the cell proliferation behavior. The structural effects on NRAS protein regarding these five mutations with the highest binding affinity to GTP were analyzed using the HOPE server. All these mutations were shown to lead to defects in NRAS structure and function.

Exploring the thermodynamic stability of top-docked GTP-NRAS complexes through molecular dynamics simulations showed higher stability and compactness profiles for the SNP models, particularly for models #2, #6, and #16, as compared to the native holo protein. This was obvious by depicting steadier and lower average valued trajectories for alpha-carbon RMSDs, Rg, and SASA. Depicting steady, rapidly equilibrated, and low-valued RMSD tones is associated with a protein’s ternary structure stability and excellent ligand-pocket accommodation, particularly when the ligand RMSDs are lower than their respective protein’s tones (Arnittali et al., 2019). The latter is based on the fact that the RMSD trajectory-based analytical parameter is indicative of structural distance deviations for atom groups (protein or ligand) in relation to their reference coordinates (Schreiner et al., 2012). Both Rg and SASA trajectories emphasized the preferential higher stability and structural compactness/tightness of the simulated SNP models #2, #6, and #16 as compared to other SNP as well as the inferior native holo protein. A protein’s SASA parameters quantify the extent of protein/solvent interactions being correlated to the solvent-accessible molecular surface for the surrounding solvent (Pirolli et al., 2014). Decreased SASA tones imply relative structural shrinkage under the influence of the solvent’s surface charges, providing more compact/stable conformations. On similar bases, lower Rgs reflect sustained structural compactness and stability since this parameter defines the mass-weighted RMSD for atom groups in relation to their common mass center (Likić et al., 2005). It is worth noting that the abovementioned thermodynamic stable behavior, evaluated through RMSDs, Rgs, and SASAs, confers the complex’s structural convergence within the designated 100-ns window as being sufficient with no need for a further time extension (Soltan et al., 2022; Zaki et al., 2022).

Validation of SNP model stability was also confirmed through monitoring the ΔRMSF tones across the molecular dynamics-simulated trajectories. This analytical parameter infers the protein amino acids’ average deviation from their reference position, providing information regarding the protein’s inherited flexibility/mobility down to their constituting amino acid level (Benson and Daggett, 2012). Higher immobility/stability profiles for the GTP-binding and Mg^2+^-coordination motifs within the top-stable SNP models were also consistent with the protein/ligand RMSDs, Rgs, and SASAs, inferring preferential GTP-binding. The most interesting finding within the ΔRMSF analysis is the consistent flexible residue range 37–50 in the Gly13 SNP model as well as the native holo proteins. This residue range constitutes most of the switch-I residues, which highlighted a significant conformational/orientational shift across the molecular dynamics simulations. This was confirmed throughout the conformational analysis, where that switch-I loop attained an open state at the end of the simulation runs. As being reported for GTPase members, the switch-I could interconvert between a closed conformation, associated with effector protein binding, and an opened state, being significant for nucleotide exchange (Kalbitzer and Spoerner, 2013; Liao et al., 2008). Inherited flexibility of the NRAS switch-I domain was also confirmed through the herein presented normal mode analysis depicting higher B-factor values and deformability. Preferential residue-wise inherited flexibility for the Gly13 SNP models was also highlighted throughout the furnished normal mode analysis, depicting higher eigenvalues than other GNP and native proteins. Another interesting finding is that the Gly13 SNP models also depicted particularly highly flexible residues at switch-II (Tyr64, Ser65, Ala66) conferring their conformational/orientation alterations. Conformational analysis translated this switch-II dynamic behavior as being related to a shift from the T-state to the F state, where the latter is reported to be catalytically active (Tetlow and Tamanoi, 2013). Depicting non-significant T-to-F state shift for the native holo NRAS and models #6 and #16 was further confirmed by having these switch-II residues with lower negative or even positive ΔRMSF values. Our findings suggest a different conformational state for the SNP models #1, #2, and #3 that could favor relevant significant GTP catalysis and nucleotide exchange in relation to the native NRAS conformer.

Applying the molecular dynamics based MM-PBSA free binding energy calculations was beneficial for highlighting the nature of GTP-NRAS binding as well as pinpointing the hot-spot residues important for GTP-binding. MM-PBSA accurately accounts for ligand-protein affinity better than static or even most sophisticated flexible molecular docking. It is considered of comparable accuracy to free energy perturbation, yet with significant cost-effective computational expenses (Kumari et al., 2014a). Moreover, the binding-free energies of the SNP models signified the role of SNP residues for GTP anchoring and binding as depicted by high residue-wise energy contributions compared to native amino acids. This was consistent with the ΔRMSF findings as these SNP residues were also with high inflexibility profiles (positive values). In terms of energy, the arginine SNP residues contributed to the increase in the Coulomb’s electrostatic potentials, favoring GTP-binding at models #1 and #6. The significance of ΔG Electrostatic for the more hydrophilic SNP models was further confirmed through VMD hydrogen–bond analysis since both models depicted the widest range of hydrophilic interactions with NRAS polar residues. Nevertheless, the latter ΔG Electrostatic preferentiality was with significant cost since the polar solvation energies were also higher than in other simulated models. This could impose a penalty against GTP since the ligand binding is considered a solvent substitution process (Zou et al., 1999). This was confirmed since the lipophilic G13V SNP residues achieved a lower polar solvation penalty. On the other hand, the G13C SNP residue was depicted as being more beneficial for GTP anchoring since the polar nonionized cysteine residue satisfied the polar character of the GTP molecule through higher ΔG Electrostatic without compromising the binding affinity via increasing the repulsive polar solvation energies. The lipophilic SNP V152F was considered significant for improving the hydrophobic potentials without significant energy penalties. All the above MM-PBSA free binding calculations recapitulated the preliminary docking results, where SNP models have a higher affinity toward GTP-binding than the native protein.

PTMs were analyzed using the MusiteDeep server; C164, I55R, and Y64 mutations were found to affect PTMs of NRAS. PTMs could modulate the functions of proteins and affect all features of their biology, including cellular localization, stability, and interaction with co-factors (Brunmeir and Xu, 2018). Therefore, affecting PTMs could lead to significant impacts on protein function. The confirmed existence of interactions among diverse genetic loci showed the importance of studying gene–gene interactions in the analysis of disease–gene associations (Cordell, 2009). The GeneMANIA tool revealed that the *SHOC2* gene showed the strongest interaction with the *NRAS* gene, followed by the STK19 gene and RGS12 gene consecutively. *NRAS* variants could have an effect on these closely connected genes to *NRAS* as well.

Overall, 17 mutations were found to be the most deleterious mutations in NRAS protein. Among these SNPs, G60E, S145L, and R164C were found to have the lowest risk, as S145L did not show a decreasing effect on protein stability and did not show high affinity to GTP and R164C was found to be located on an intermediately conserved site and was not located on the important small GTP-binding protein domain. G60E showed the lowest binding affinity to GTP. All other 14 mutations were expected to affect NRAS structure and function and were expected to have a role in increasing carcinogenesis. In addition, the five mutations that showed the highest binding affinity to GTP (G13R, G13C, G13V, P34R, and V152F) are expected to have the highest risk of carcinogenesis. It is important to mention that some of the nominated deleterious SNPs in the current study have already been correlated to carcinogenesis in previous wet-lab studies, where G13 mutations have been associated with chronic myelomonocytic leukemia and juvenile myelomonocytic leukemia progression in humans and mice (Kong et al., 2016) and G13V mutation was found to cause an aggressive phenotype with anaplastic transformation in thyroid cancer (Pozdeyev et al., 2018); this mutation was also found in metastatic colorectal cancer along with BRAF codons 594 and 596 mutations (Cremolini et al., 2015). Other mutations that were predicted to be deleterious from our analysis are G60E, S39F, and Y64D, and these mutations have been correlated with myeloid leukemia, oral mucosal melanoma, and primary acral melanoma patients, respectively (Tyner et al., 2009; Chen et al., 2018; Moon et al., 2018). On the other hand, mutations such as G12 and Q61 have been correlated with leukemia patients (Kong et al., 2016), and the current study failed to nominate them among the deleterious candidates. Therefore, a thorough investigation of our filtered mutations through experimental methods is an essential next step.

## Conclusion

The NRAS protein has a vital role in regulating cell proliferation, motility, and apoptosis. It has a domain that interacts with a GTP molecule and converts it to GDP to control the cell activity status. Hence, the conservation and structural conformation of this domain are highly important for the protein functional role. The current computational study sheds light on the deleterious effects of the nsSNPs on the structural and functional characteristics of this protein. Our study demonstrated that 14 SNPs are predicted to affect NRAS structure and function, where the 5 mutations that showed the highest binding affinity to GTP (G13R, G13C, G13V, P34R, and V152F) were nominated as the highest risky ones. Thermodynamic stability was assured for these top-docked SNP proteins through molecular dynamics simulations. Furthermore, the Gly13 SNP model adopted differential conformational states that could confer significant GTP catalysis and nucleotide exchange. As *NRAS* mutations have been linked to several forms of tumors in many previous studies, the current analysis would represent a guide for future experimental SNP validation and clinical analysis on a large group of people.

## Data Availability

The original contributions presented in the study are included in the article/Supplementary Material; further inquiries can be directed to the corresponding authors.

## References

[B1] Adzhubei I. A. Schmidt S. Peshkin L. Ramensky V. E. Gerasimova A. Bork P. (2010). A Method and Server for Predicting Damaging Missense Mutations. Nat. Methods 7, 248–249. 10.1038/nmeth0410-248 20354512 PMC2855889

[B2] Albuquerque S. O. Barros T. G. Dias L. R. S. Lima C. H. d. S. Azevedo P. H. R. d. A. Flores-Junior L. A. P. (2020). Biological Evaluation and Molecular Modeling of Peptidomimetic Compounds as Inhibitors for O-GlcNAc Transferase (OGT). Eur. J. Pharm. Sci. 154, 105510. 10.1016/j.ejps.2020.105510 32801002

[B3] Araki M. Shima F. Yoshikawa Y. Muraoka S. Ijiri Y. Nagahara Y. (2011). Solution Structure of the State 1 Conformer of GTP-Bound H-Ras Protein and Distinct Dynamic Properties between the State 1 and State 2 Conformers. J. Biol. Chem. 286, 39644–39653. 10.1074/jbc.M111.227074 21930707 PMC3234787

[B4] Arnittali M. Rissanou A. N. Harmandaris V. (2019). Structure of Biomolecules through Molecular Dynamics Simulations. Procedia Comput. Sci. 156, 69–78. 10.1016/j.procs.2019.08.181

[B5] Ashkenazy H. Abadi S. Martz E. Chay O. Mayrose I. Pupko T. (2016). ConSurf 2016: an Improved Methodology to Estimate and Visualize Evolutionary Conservation in Macromolecules. Nucleic Acids Res. 44, W344–W350. 10.1093/nar/gkw408 27166375 PMC4987940

[B6] Bava K. A. Gromiha M. M. Uedaira H. Kitajima K. Sarai A. (2004). ProTherm, Version 4.0: Thermodynamic Database for Proteins and Mutants. Nucleic Acids Res. 32, 120D–121D. 10.1093/nar/gkh082 PMC308816 14681373

[B7] Benson N. C. Daggett V. (2012). A Comparison of Multiscale Methods for the Analysis of Molecular Dynamics Simulations. J. Phys. Chem. B 116, 8722–8731. 10.1021/jp302103t 22494262 PMC3406285

[B8] Berezin C. Glaser F. Rosenberg J. Paz I. Pupko T. Fariselli P. (2004). ConSeq: the Identification of Functionally and Structurally Important Residues in Protein Sequences. Bioinformatics 20, 1322–1324. 10.1093/bioinformatics/bth070 14871869

[B9] Bivona T. G. Quatela S. E. Bodemann B. O. Ahearn I. M. Soskis M. J. Mor A. (2006). PKC Regulates a Farnesyl-Electrostatic Switch on K-Ras that Promotes its Association with Bcl-XL on Mitochondria and Induces Apoptosis. Mol. Cell. 21, 481–493. 10.1016/j.molcel.2006.01.012 16483930

[B10] Blum M. Chang H.-Y. Chuguransky S. Grego T. Kandasaamy S. Mitchell A. (2021). The InterPro Protein Families and Domains Database: 20 Years on. Nucleic Acids Res. 49, D344–d354. 10.1093/nar/gkaa977 33156333 PMC7778928

[B11] Bronte G. Silvestris N. Castiglia M. Galvano A. Passiglia F. Sortino G. (2015). New Findings on Primary and Acquired Resistance to Anti-EGFR Therapy in Metastatic Colorectal Cancer: Do All Roads Lead to RAS? Oncotarget 6, 24780–24796. 10.18632/oncotarget.4959 26318427 PMC4694794

[B12] Brunmeir R. Xu F. (2018). Functional Regulation of PPARs through Post-Translational Modifications. Ijms 19, 1738. 10.3390/ijms19061738 PMC6032173 29895749

[B13] Capriotti E. Calabrese R. Casadio R. (2006). Predicting the Insurgence of Human Genetic Diseases Associated to Single Point Protein Mutations with Support Vector Machines and Evolutionary Information. Bioinformatics 22, 2729–2734. 10.1093/bioinformatics/btl423 16895930

[B14] Capriotti E. Calabrese R. Fariselli P. Martelli P. Altman R. B. Casadio R. (2013). WS-SNPs&GO: a Web Server for Predicting the Deleterious Effect of Human Protein Variants Using Functional Annotation. BMC Genomics 14 (Suppl. 3), S6. 10.1186/1471-2164-14-s3-s6 PMC3665478 23819482

[B15] Capriotti E. Fariselli P. Casadio R. (2005). I-Mutant2.0: Predicting Stability Changes upon Mutation from the Protein Sequence or Structure. Nucleic Acids Res. 33, W306–W310. 10.1093/nar/gki375 15980478 PMC1160136

[B16] Chasman D. Adams R. M. (2001). Predicting the Functional Consequences of Non-synonymous Single Nucleotide Polymorphisms: Structure-Based Assessment of Amino Acid variation11Edited by F. Cohen. J. Mol. Biol. 307, 683–706. 10.1006/jmbi.2001.4510 11254390

[B17] Chen C.-C. Er T.-K. Liu Y.-Y. Hwang J.-K. Barrio M. J. Rodrigo M. (2013). Computational Analysis of KRAS Mutations: Implications for Different Effects on the KRAS p.G12D and p.G13D Mutations. PLoS One 8, e55793. 10.1371/journal.pone.0055793 23437064 PMC3577811

[B18] Chen F. Zhang Q. Wang Y. Wang S. Feng S. Qi L. (2018). *Kit, NRAS, BRAF* and *FMNL2* Mutations in Oral Mucosal Melanoma and a Systematic Review of the Literature. Oncol. Lett. 15 (6), 9786–9792. 10.3892/ol.2018.8558 29805686 PMC5958699

[B19] Cheng J. Randall A. Baldi P. (2006). Prediction of Protein Stability Changes for Single-Site Mutations Using Support Vector Machines. Proteins 62, 1125–1132. 10.1002/prot.20810 16372356

[B20] Chiu V. K. Bivona T. Hach A. Sajous J. B. Silletti J. Wiener H. (2002). Ras Signalling on the Endoplasmic Reticulum and the Golgi. Nat. Cell. Biol. 4, 343–350. 10.1038/ncb783 11988737

[B21] Choi Y. Chan A. P. (2015). PROVEAN Web Server: a Tool to Predict the Functional Effect of Amino Acid Substitutions and Indels. Bioinformatics 31, 2745–2747. 10.1093/bioinformatics/btv195 25851949 PMC4528627

[B22] Cordell H. J. (2009). Detecting Gene-Gene Interactions that Underlie Human Diseases. Nat. Rev. Genet. 10, 392–404. 10.1038/nrg2579 19434077 PMC2872761

[B23] Cremolini C. Di Bartolomeo M. Amatu A. Antoniotti C. Moretto R. Berenato R. (2015). BRAF Codons 594 and 596 Mutations Identify a New Molecular Subtype of Metastatic Colorectal Cancer at Favorable Prognosis. Ann. Oncol. 26, 2092–2097. 10.1093/annonc/mdv290 26153495

[B24] Dakal T. C. Kala D. Dhiman G. Yadav V. Krokhotin A. Dokholyan N. V. (2017). Predicting the Functional Consequences of Non-synonymous Single Nucleotide Polymorphisms in IL8 Gene. Sci. Rep. 7, 6525. 10.1038/s41598-017-06575-4 28747718 PMC5529537

[B25] Damani Shah H. Saranath D. Das S. Kharkar P. Karande A. (2019). In‐silico Identification of Small Molecules Targeting H‐Ras and In‐vitro Cytotoxicity with Caspase‐mediated Apoptosis in Carcinoma Cells. J Cell. Biochem. 120, 5519–5530. 10.1002/jcb.27836 30367521

[B26] Deller M. C. Kong L. Rupp B. (2016). Protein Stability: a Crystallographer's Perspective. Acta Cryst. Sect. F. 72, 72–95. 10.1107/S2053230X15024619 PMC4741188 26841758

[B27] Dhar S. Mridha S. Bhattacharjee P. (2022). Mutational Landscape Screening through Comprehensive In Silico Analysis for Polycystic Ovarian Syndrome-Related Genes. Reprod. Sci. 29, 480–496. 10.1007/s43032-021-00752-7 34697776

[B28] Eisfeld A.-K. Schwind S. Hoag K. W. Walker C. J. Liyanarachchi S. Patel R. (2014). NRAS Isoforms Differentially Affect Downstream Pathways, Cell Growth, and Cell Transformation. Proc. Natl. Acad. Sci. U.S.A. 111, 4179–4184. 10.1073/pnas.1401727111 24586049 PMC3964043

[B29] Elhady S. S. Abdelhameed R. F. A. Malatani R. T. Alahdal A. M. Bogari H. A. Almalki A. J. (2021). Molecular Docking and Dynamics Simulation Study of Hyrtios Erectus Isolated Scalarane Sesterterpenes as Potential Sars-Cov-2 Dual Target Inhibitors. Biology 10, 389. 10.3390/biology10050389 34062724 PMC8147222

[B30] Ellerhorst J. A. Greene V. R. Ekmekcioglu S. Warneke C. L. Johnson M. M. Cooke C. P. (2011). Clinical Correlates ofNRASandBRAFMutations in Primary Human Melanoma. Clin. Cancer Res. 17, 229–235. 10.1158/1078-0432.CCR-10-2276 20975100 PMC3022950

[B31] Emadi E. Akhoundi F. Kalantar S. M. Emadi-Baygi M. (2020). Predicting the Most Deleterious Missense nsSNPs of the Protein Isoforms of the Human HLA-G Gene and In Silico Evaluation of Their Structural and Functional Consequences. BMC Genet. 21, 94. 10.1186/s12863-020-00890-y 32867672 PMC7457528

[B32] Fehrenbacher N. Bar-Sagi D. Philips M. (2009). Ras/MAPK Signaling from Endomembranes. Mol. Oncol. 3, 297–307. 10.1016/j.molonc.2009.06.004 19615955 PMC3003591

[B33] Geourjon C. Deléage G. (1995). SOPMA: Significant Improvements in Protein Secondary Structure Prediction by Consensus Prediction from Multiple Alignments. Bioinformatics 11, 681–684. 10.1093/bioinformatics/11.6.681 8808585

[B34] Gharib A. F. Eldeen M. A. Khalifa A. S. Elsawy W. H. Eed E. M. Askary A. El. (2021). Assessment of Glutathione Peroxidase-1 ( GPX1 ) Gene Expression as a Specific Diagnostic and Prognostic Biomarker in Malignant Pleural Mesothelioma. Diagn. (Basel) 11, 2285. 10.3390/diagnostics11122285 PMC8700378 34943522

[B35] Giannou A. D. Marazioti A. Kanellakis N. I. Giopanou I. Lilis I. Zazara D. E. (2017). NRAS Destines Tumor Cells to the Lungs. EMBO Mol. Med. 9, 672–686. 10.15252/emmm.201606978 28341702 PMC5697015

[B36] Hecht M. Bromberg Y. Rost B. (2015). Better Prediction of Functional Effects for Sequence Variants. BMC Genomics 16 (Suppl. 8), S1. 10.1186/1471-2164-16-s8-s1 PMC4480835 26110438

[B37] Hossain M. S. Roy A. S. Islam M. S. (2020). In Silico analysis Predicting Effects of Deleterious SNPs of Human RASSF5 Gene on its Structure and Functions. Sci. Rep. 10, 14542. 10.1038/s41598-020-71457-1 32884013 PMC7471297

[B38] Jakob J. A. Bassett R. L. Jr Ng C. S. Curry J. L. Joseph R. W. Alvarado G. C. (2012). NRAS Mutation Status Is an Independent Prognostic Factor in Metastatic Melanoma. Cancer 118, 4014–4023. 10.1002/cncr.26724 22180178 PMC3310961

[B39] Jia M. Yang B. Li Z. Shen H. Song X. Gu W. (2014). Computational Analysis of Functional Single Nucleotide Polymorphisms Associated with the CYP11B2 Gene. PLoS One 9, e104311. 10.1371/journal.pone.0104311 25102047 PMC4125216

[B40] Johnson C. W. Reid D. Parker J. A. Salter S. Knihtila R. Kuzmic P. (2017). The Small GTPases K-Ras, N-Ras, and H-Ras Have Distinct Biochemical Properties Determined by Allosteric Effects. J. Biol. Chem. 292, 12981–12993. 10.1074/jbc.M117.778886 28630043 PMC5546037

[B41] Kalbitzer H. R. Spoerner M. (2013). State 1(T) Inhibitors of Activated Ras. Enzymes 33 Pt A, 69–94. 10.1016/B978-0-12-416749-0.00004-X 25033801

[B42] Kong G. Chang Y.-I. You X. Ranheim E. A. Zhou Y. Burd C. E. (2016). The Ability of Endogenous Nras Oncogenes to Initiate Leukemia Is Codon-dependent. Leukemia 30, 1935–1938. 10.1038/leu.2016.89 27109513 PMC5347394

[B43] Kumar S. Sharma D. Narasimhan B. Ramasamy K. Shah S. A. A. Lim S. M. (2019). Computational Approaches: Discovery of GTPase HRas as Prospective Drug Target for 1,3-diazine Scaffolds. BMC Chem. 13, 96. 10.1186/s13065-019-0613-8 31355369 PMC6659553

[B44] Kumar S. Singh J. Narasimhan B. Shah S. A. A. Lim S. M. Ramasamy K. (2018). Reverse Pharmacophore Mapping and Molecular Docking Studies for Discovery of GTPase HRas as Promising Drug Target for Bis-Pyrimidine Derivatives. Chem. Central J. 12, 106. 10.1186/s13065-018-0475-5 PMC6768019 30345469

[B45] Kumari R. Kumar R. Consortium O. S. D. D. Lynn A. (2014a). g_mmpbsa--A GROMACS Tool for MM-PBSA and its Optimization for High-Throughput Binding Energy Calculations. J. Chem. Inf. Model. 54 (7), 1951–1962. 10.1021/ci500020m 24850022

[B46] Kumari R. Kumar R. Lynn A. (2014b). g_mmpbsa-A GROMACS Tool for High-Throughput MM-PBSA Calculations. J. Chem. Inf. Model. 54, 1951–1962. 10.1021/ci500020m 24850022

[B47] Kwong L. N. Costello J. C. Liu H. Jiang S. Helms T. L. Langsdorf A. E. (2012). Oncogenic NRAS Signaling Differentially Regulates Survival and Proliferation in Melanoma. Nat. Med. 18, 1503–1510. 10.1038/nm.2941 22983396 PMC3777533

[B48] Liao J. Shima F. Araki M. Ye M. Muraoka S. Sugimoto T. (2008). Two Conformational States of Ras GTPase Exhibit Differential GTP-Binding Kinetics. Biochem. Biophysical Res. Commun. 369, 327–332. 10.1016/j.bbrc.2008.01.169 18291096

[B49] Liao P.-Y. Lee K. H. (2010). From SNPs to Functional Polymorphism: The Insight into Biotechnology Applications. Biochem. Eng. J. 49, 149–158. 10.1016/j.bej.2009.12.021

[B50] Likić V. A. Gooley P. R. Speed T. P. Strehler E. E. (2005). A Statistical Approach to the Interpretation of Molecular Dynamics Simulations of Calmodulin Equilibrium Dynamics. Protein Sci. 14, 2955–2963. 10.1110/ps.051681605 16322577 PMC2253239

[B51] López-Blanco J. R. Aliaga J. I. Quintana-Ortí E. S. Chacón P. (2014). IMODS: Internal Coordinates Normal Mode Analysis Server. Nucleic Acids Res. 42, W271–W276. 10.1093/nar/gku339 24771341 PMC4086069

[B52] Matallanas D. Sanz-Moreno V. Arozarena I. Calvo F. Agudo-Ibáñez L. Santos E. (2006). Distinct Utilization of Effectors and Biological Outcomes Resulting from Site-specific Ras Activation: Ras Functions in Lipid Rafts and Golgi Complex Are Dispensable for Proliferation and Transformation. Mol. Cell. Biol. 26, 100–116. 10.1128/mcb.26.1.100-116.2006 16354683 PMC1317613

[B53] Moon K. R. Choi Y. D. Kim J. M. Jin S. Shin M.-H. Shim H.-J. (2018). Genetic Alterations in Primary Acral Melanoma and Acral Melanocytic Nevus in Korea: Common Mutated Genes Show Distinct Cytomorphological Features. J. Investigative Dermatology 138, 933–945. 10.1016/j.jid.2017.11.017 29191620

[B54] Muñoz-Maldonado C. Zimmer Y. Medová M. (2019). A Comparative Analysis of Individual Ras Mutations in Cancer Biology. Front. Oncol. 9. 10.3389/fonc.2019.01088 PMC6813200 31681616

[B55] Nussinov R. Zhang M. Maloney R. Jang H. (2021). Ras Isoform-specific Expression, Chromatin Accessibility, and Signaling. Biophys. Rev. 13, 489–505. 10.1007/s12551-021-00817-6 34466166 PMC8355297

[B56] Ohashi K. Sequist L. V. Arcila M. E. Lovly C. M. Chen X. Rudin C. M. (2013). Characteristics of Lung Cancers Harboring NRAS Mutations. Clin. Cancer Res. 19, 2584–2591. 10.1158/1078-0432.CCR-12-3173 23515407 PMC3643999

[B57] Oleg T. Arthur J., O. (2010). AutoDock Vina: Improving the Speed and Accuracy of Docking with a New Scoring Function, Efficient Optimization, and Multithreading. J. Comput. Chem. 31 (2), 455–461. 10.1002/jcc.21334 19499576 PMC3041641

[B58] Omerovic J. Laude A. J. Prior I. A. (2007). Ras Proteins: Paradigms for Compartmentalised and Isoform-specific Signalling. Cell. Mol. Life Sci. 64, 2575–2589. 10.1007/s00018-007-7133-8 17628742 PMC2561238

[B59] Páll S. Abraham M. J. Kutzner C. Hess B. Lindahl E. (2015). “Tackling Exascale Software Challenges in Molecular Dynamics Simulations with GROMACS,” in Lecture Notes in Computer Science, Springer, Cham, 3–27. (including subseries Lecture Notes in Artificial Intelligence and Lecture Notes in Bioinformatics). 10.1007/978-3-319-15976-8_1

[B60] Parker J. A. Mattos C. (2015). The Ras-Membrane Interface: Isoform-specific Differences in the Catalytic Domain. Mol. Cancer Res. 13, 595–603. 10.1158/1541-7786.MCR-14-0535 25566993

[B61] Patnala R. Clements J. Batra J. (2013). Candidate Gene Association Studies: a Comprehensive Guide to Useful in Silicotools. BMC Genet. 14, 39. 10.1186/1471-2156-14-39 23656885 PMC3655892

[B62] Pirolli D. Sciandra F. Bozzi M. Giardina B. Brancaccio A. De Rosa M. C. (2014). Insights from Molecular Dynamics Simulations: Structural Basis for the V567D Mutation-Induced Instability of Zebrafish Alpha-Dystroglycan and Comparison with the Murine Model. PLoS One 9, e103866. 10.1371/journal.pone.0103866 25078606 PMC4117597

[B63] Pozdeyev N. Gay L. M. Sokol E. S. Hartmaier R. Deaver K. E. Davis S. (2018). Genetic Analysis of 779 Advanced Differentiated and Anaplastic Thyroid Cancers. Clin. Cancer Res. 24, 3059–3068. 10.1158/1078-0432.CCR-18-0373 29615459 PMC6030480

[B64] Purushothaman B. Suganthi N. Jothi A. Shanmugam K. (2019). Molecular Docking Studies of Potential Anticancer Agents from Ocimum Basilicum L. Against Human Colorectal Cancer Regulating Genes: An Insilico Approach. Rese. Jour. Pharm. Technol. 12, 3423. 10.5958/0974-360x.2019.00579.1

[B65] Rajput N. Gahlay G. K. (2021). Identification and In Silico Characterization of Deleterious Single Nucleotide Variations in Human ZP2 Gene. Front. Cell. Dev. Biol. 9. 10.3389/fcell.2021.763166 PMC8635754 34869353

[B66] Rocks O. Peyker A. Bastiaens P. I. (2006). Spatio-temporal Segregation of Ras Signals: One Ship, Three Anchors, Many Harbors. Curr. Opin. Cell. Biol. 18, 351–357. 10.1016/j.ceb.2006.06.007 16781855

[B67] Schreiner W. Karch R. Knapp B. Ilieva N. (2012). Relaxation Estimation of RMSD in Molecular Dynamics Immunosimulations. Comput. Math. Methods Med. 2012, 1–9. 10.1155/2012/173521 PMC3457668 23019425

[B68] Seeliger D. De Groot B. L. (2010). Ligand Docking and Binding Site Analysis with PyMOL and Autodock/Vina. J. Comput. Aided. Mol. Des. 24, 417–422. 10.1007/s10822-010-9352-6 20401516 PMC2881210

[B69] Sim N.-L. Kumar P. Hu J. Henikoff S. Schneider G. Ng P. C. (2012). SIFT Web Server: Predicting Effects of Amino Acid Substitutions on Proteins. Nucleic Acids Res. 40, W452–W457. 10.1093/nar/gks539 22689647 PMC3394338

[B70] Soltan M. A. Behairy M. Y. Abdelkader M. S. Albogami S. Fayad E. Eid R. A. (2022). In Silico Designing of an Epitope-Based Vaccine against Common *E. coli* Pathotypes. Front. Med. 9, 1–22. 10.3389/fmed.2022.829467 PMC8931290 35308494

[B71] Soltan M. A. Eldeen M. A. Elbassiouny N. Kamel H. L. Abdelraheem K. M. El-Gayyed H. A. (2021). In Silico designing of a Multitope Vaccine against Rhizopus Microsporus with Potential Activity against Other Mucormycosis Causing Fungi. Cells 10, 3014. 10.3390/cells10113014 34831237 PMC8616407

[B72] Sun G. Su G. Liu F. Han W. (2019). NRAS Contributes to Retinoblastoma Progression through SNHG16/miR-183-5p/NRAS Regulatory Network. Ott Vol. 12, 10703–10715. 10.2147/OTT.S232470 PMC6902855 31827328

[B73] Tan H. (2017). The Association between Gene SNPs and Cancer Predisposition: Correlation or Causality? EBioMedicine 16, 8–9. 10.1016/j.ebiom.2017.01.047 28163041 PMC5474513

[B74] Tetlow A. L. Tamanoi F., and (2013). The Ras Superfamily G-Proteins. Enzymes 33 Pt A, 1–14. 10.1016/B978-0-12-416749-0.00001-4 25033798

[B75] Tyner J. W. Erickson H. Deininger M. W. N. Willis S. G. Eide C. A. Levine R. L. (2009). High-throughput Sequencing Screen Reveals Novel, Transforming RAS Mutations in Myeloid Leukemia Patients. Blood 113, 1749–1755. 10.1182/blood-2008-04-152157 19075190 PMC2647674

[B76] Venselaar H. Te Beek T. A. Kuipers R. K. Hekkelman M. L. Vriend G. (2010). Protein Structure Analysis of Mutations Causing Inheritable Diseases. An E-Science Approach with Life Scientist Friendly Interfaces. BMC Bioinforma. 11, 548. 10.1186/1471-2105-11-548 PMC2992548 21059217

[B77] Vignal A. Milan D. SanCristobal M. Eggen A. (2002). A Review on SNP and Other Types of Molecular Markers and Their Use in Animal Genetics. Genet. Sel. Evol. 34 (3), 275–305. 10.1186/1297-9686-34-3-275 12081799 PMC2705447

[B78] Voice J. K. Klemke R. L. Le A. Jackson J. H. (1999). Four Human Ras Homologs Differ in Their Abilities to Activate Raf-1, Induce Transformation, and Stimulate Cell Motility. J. Biol. Chem. 274, 17164–17170. 10.1074/jbc.274.24.17164 10358073

[B79] Wallace A. C. Laskowski R. A. Thornton J. M. (1995). LIGPLOT: a Program to Generate Schematic Diagrams of Protein-Ligand Interactions. Protein Eng. Des. Sel. 8, 127–134. 10.1093/protein/8.2.127 7630882

[B80] Wang D. Liang Y. Xu D. (2019). Capsule Network for Protein Post-translational Modification Site Prediction. Bioinformatics 35, 2386–2394. 10.1093/bioinformatics/bty977 30520972 PMC6612812

[B81] Wang D. Liu D. Yuchi J. He F. Jiang Y. Cai S. (2020). MusiteDeep: a Deep-Learning Based Webserver for Protein Post-translational Modification Site Prediction and Visualization. Nucleic Acids Res. 48, W140–w146. 10.1093/nar/gkaa275 32324217 PMC7319475

[B82] Wang D. Zeng S. Xu C. Qiu W. Liang Y. Joshi T. (2017). MusiteDeep: a Deep-Learning Framework for General and Kinase-specific Phosphorylation Site Prediction. Bioinformatics 33, 3909–3916. 10.1093/bioinformatics/btx496 29036382 PMC5860086

[B83] Warde-Farley D. Donaldson S. L. Comes O. Zuberi K. Badrawi R. Chao P. (2010). The GeneMANIA Prediction Server: Biological Network Integration for Gene Prioritization and Predicting Gene Function. Nucleic Acids Res. 38, W214–W220. 10.1093/nar/gkq537 20576703 PMC2896186

[B84] Waterhouse A. Bertoni M. Bienert S. Studer G. Tauriello G. Gumienny R. (2018). SWISS-MODEL: Homology Modelling of Protein Structures and Complexes. Nucleic Acids Res. 46, W296–W303. 10.1093/nar/gky427 29788355 PMC6030848

[B85] Wiederstein M. Sippl M. J. (2007). ProSA-web: Interactive Web Service for the Recognition of Errors in Three-Dimensional Structures of Proteins. Nucleic Acids Res. 35, W407–W410. 10.1093/nar/gkm290 17517781 PMC1933241

[B86] Yeam I. (2016). Current Advances and Prospectus of Viral Resistance in Horticultural Crops. Hortic. Environ. Biotechnol. 57, 113–122. 10.1007/s13580-016-0105-x

[B87] Yin C. Zhu B. Zhang T. Liu T. Chen S. Liu Y. (2019). Pharmacological Targeting of STK19 Inhibits Oncogenic NRAS-Driven Melanomagenesis. Cell. 176, 1113–1127. 10.1016/j.cell.2019.01.002 30712867

[B88] Zaki A. A. Ashour A. Elhady S. S. Darwish K. M. Al-Karmalawy A. A. (2022). Calendulaglycoside A Showing Potential Activity against SARS-CoV-2 Main Protease: Molecular Docking, Molecular Dynamics, and SAR Studies. J. Traditional Complementary Med. 12, 16–34. 10.1016/j.jtcme.2021.05.001 PMC8126476 34026584

[B89] Zhang J. Yang J. Zhang L. Luo J. Zhao H. Zhang J. (2020). A New SNP Genotyping Technology Target SNP-Seq and its Application in Genetic Analysis of Cucumber Varieties. Sci. Rep. 10, 5623. 10.1038/s41598-020-62518-6 32221398 PMC7101363

[B90] Zhang Y. Skolnick J. (2005). TM-align: A Protein Structure Alignment Algorithm Based on the TM-Score. Nucleic Acids Res. 33, 2302–2309. 10.1093/nar/gki524 15849316 PMC1084323

[B91] Zou X. Yaxiong Y. Kuntz I. D. (1999). Inclusion of Solvation in Ligand Binding Free Energy Calculations Using the Generalized-Born Model. J. Am. Chem. Soc. 121, 8033–8043. 10.1021/ja984102p

