# Supplementary material for: Computational Analysis of Deleterious SNPs in NRAS to Assess Their Potential Correlation With Carcinogenesis
Source: Front Genet. 2022 Aug 16;13:872845. doi: 10.3389/fgene.2022.872845 (PMC9424727; doi:10.3389/fgene.2022.872845)
Supplement: Supplementary file 1 [file DataSheet1.docx]

**Supplementary materials**

**Supplementary figures**

**
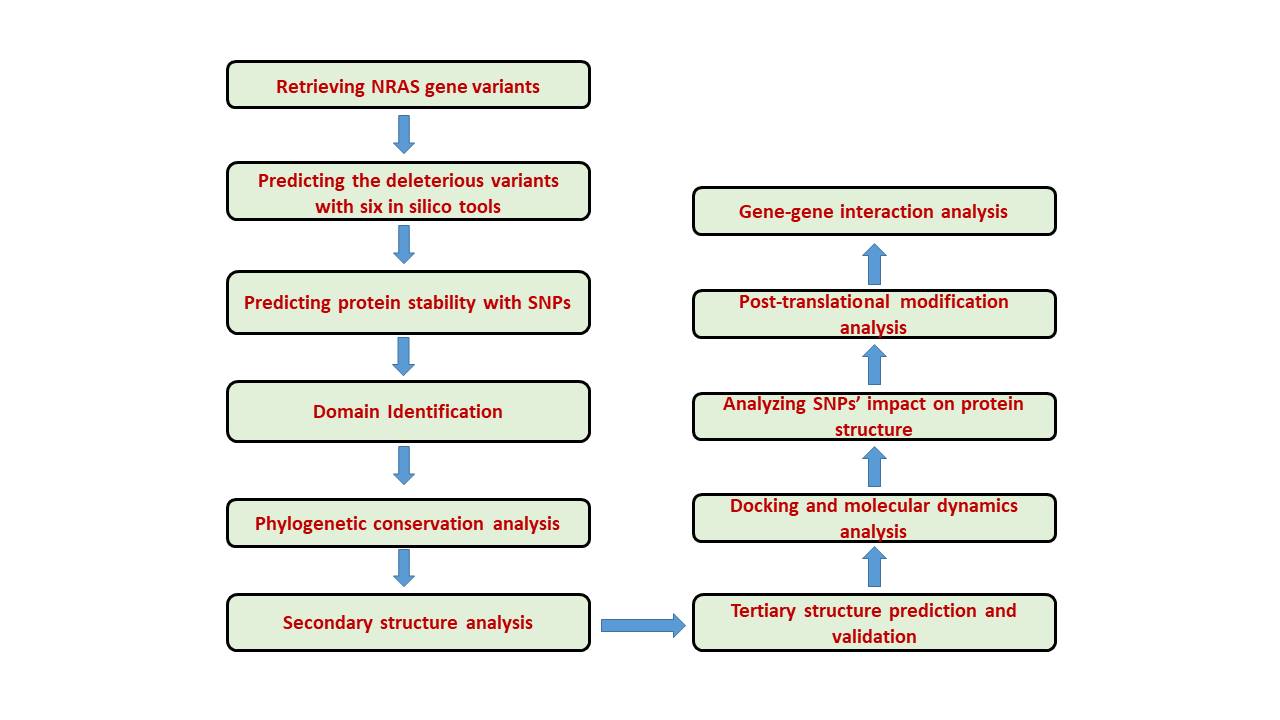
**

Supplementary figure 1. The flow of work applied in the current study.

**
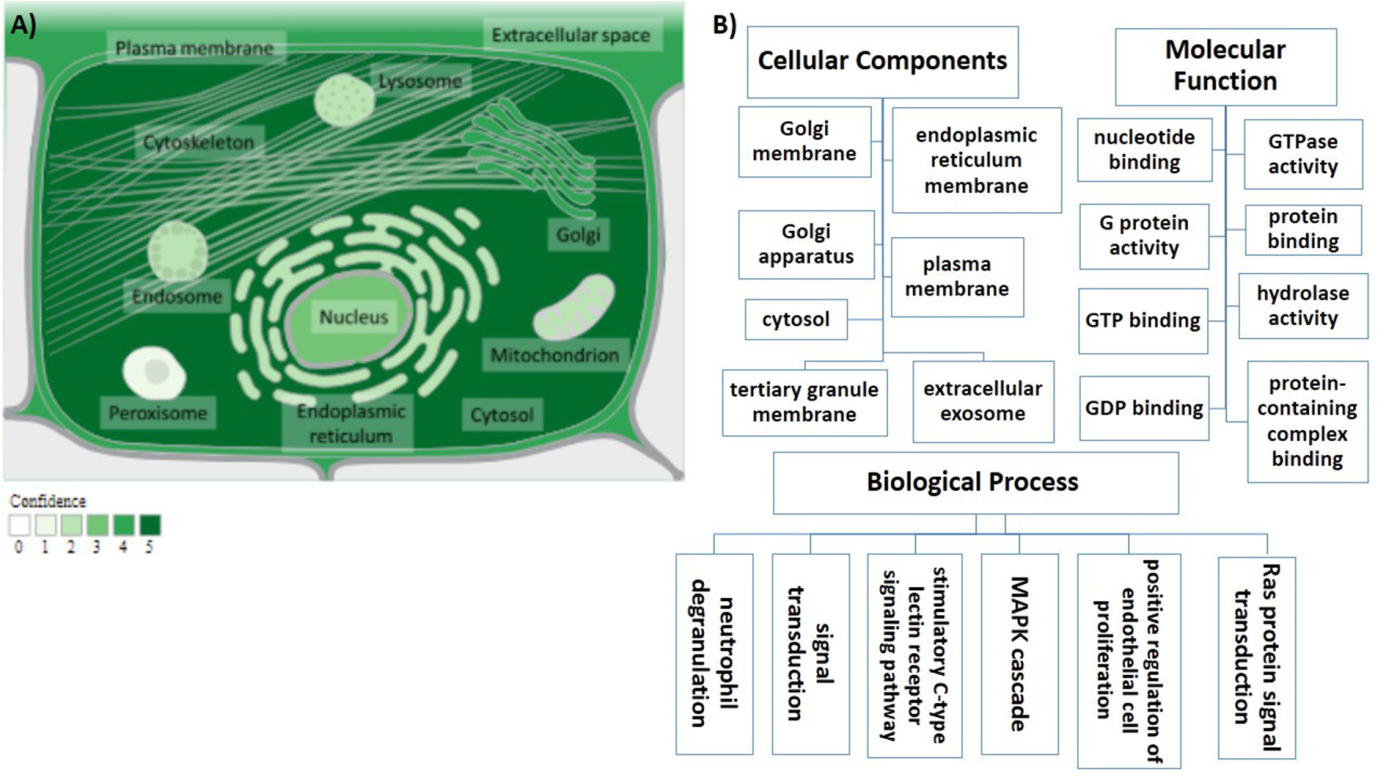
**

Supplementary figure 2. A) Subcellular localization of NRAS. The color code expresses the confidence levels with a gradient from light green expressing low confidence level to dark green expressing high confidence level (genecards.org/) with the source of the image is (Compartments.jensenlab.org/). B) Gene ontology analysis of NRAS. Cellular component, Molecular function and Biological process of NRAS are demonstrated.


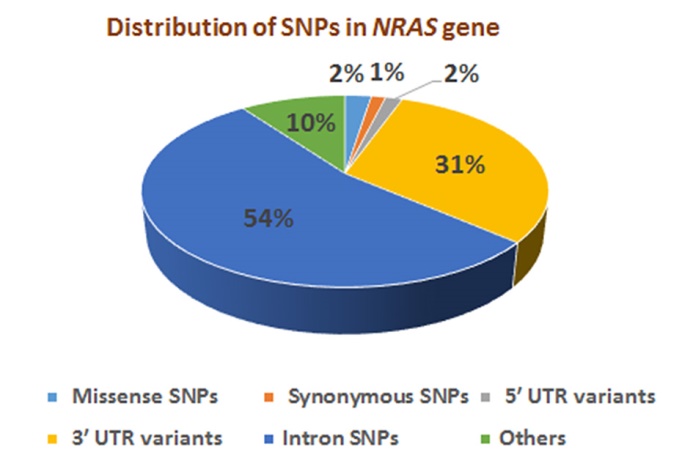


Supplementary figure 3. SNPs distribution in *NRAS* gene.

**
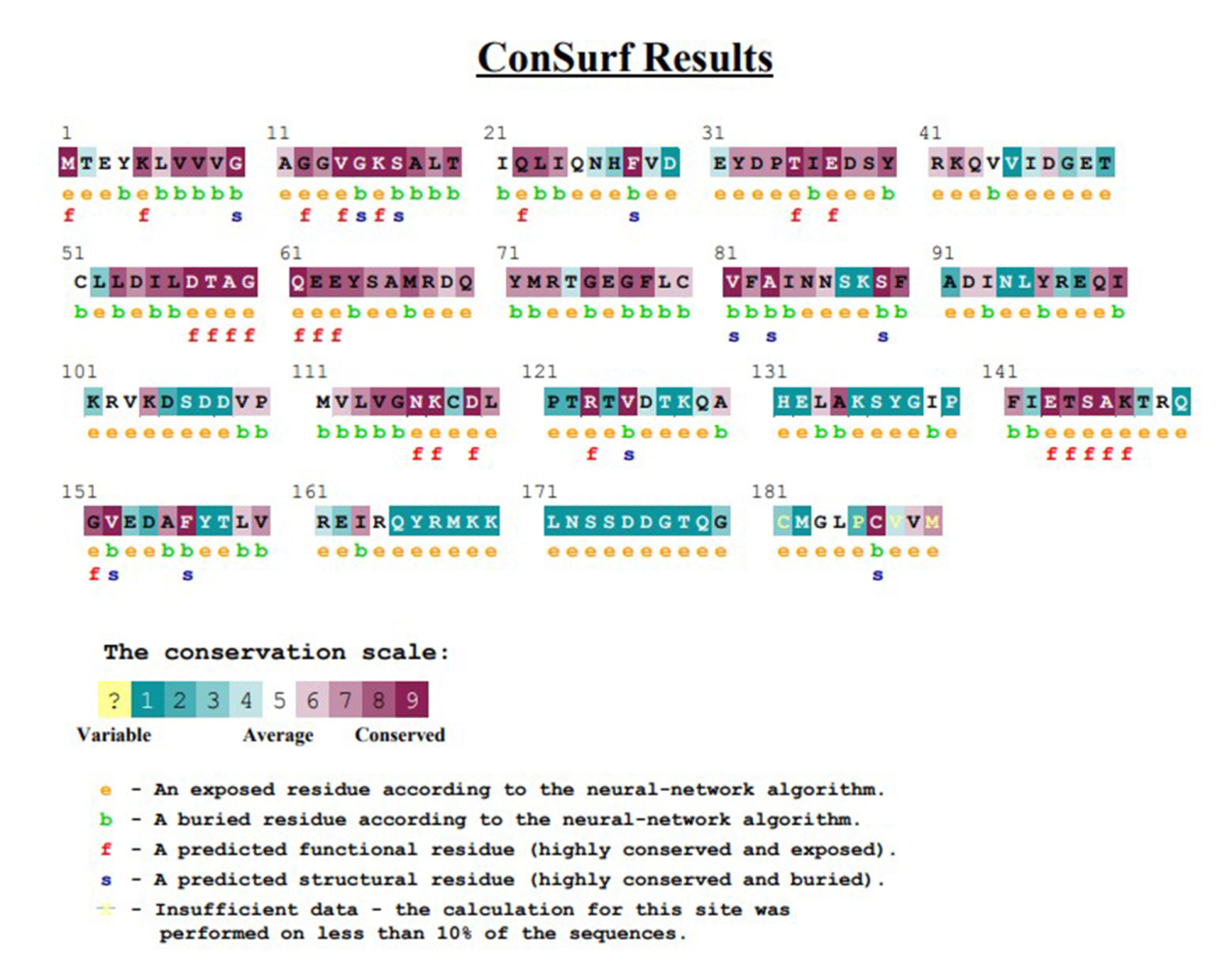
**

Supplementary figure 4. Phylogenetic conservation analysis of NRAS by ConSurf server.


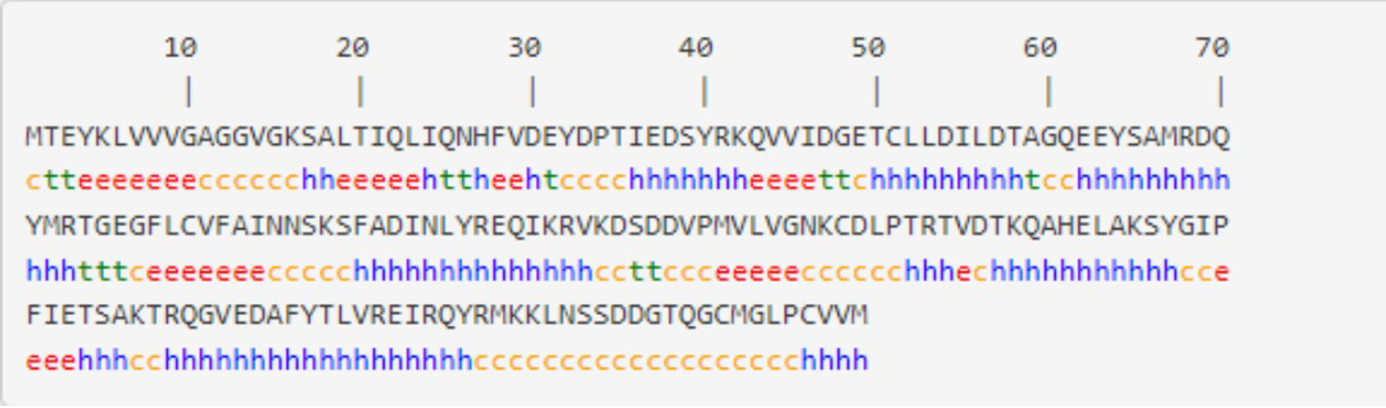


Supplementary figure 5. Analysis of NRAS Secondary structures by SOPMA tool.


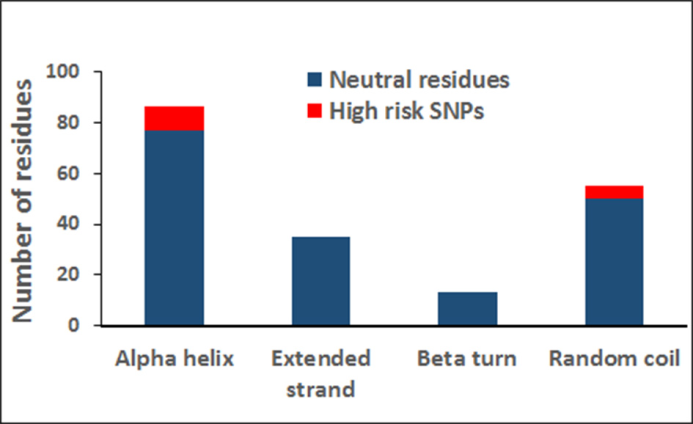


Supplementary figure 6. Distribution of NRAS sequences according to the Secondary structure with the red part of the bar indicating the deleterious missense SNPs.


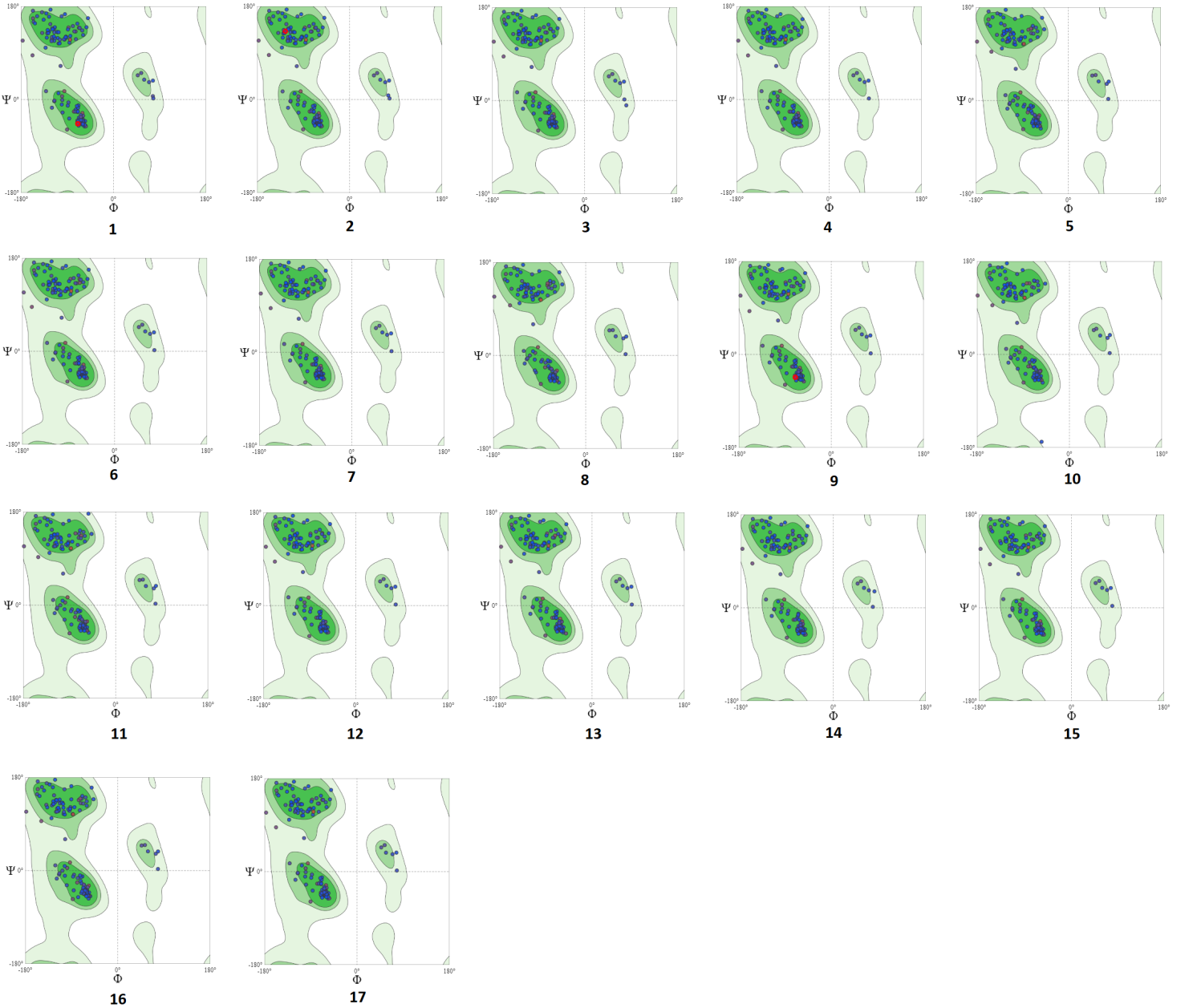


Supplementary figure 7. Ramachandran plot analysis for the designed 17 mutant proteins.


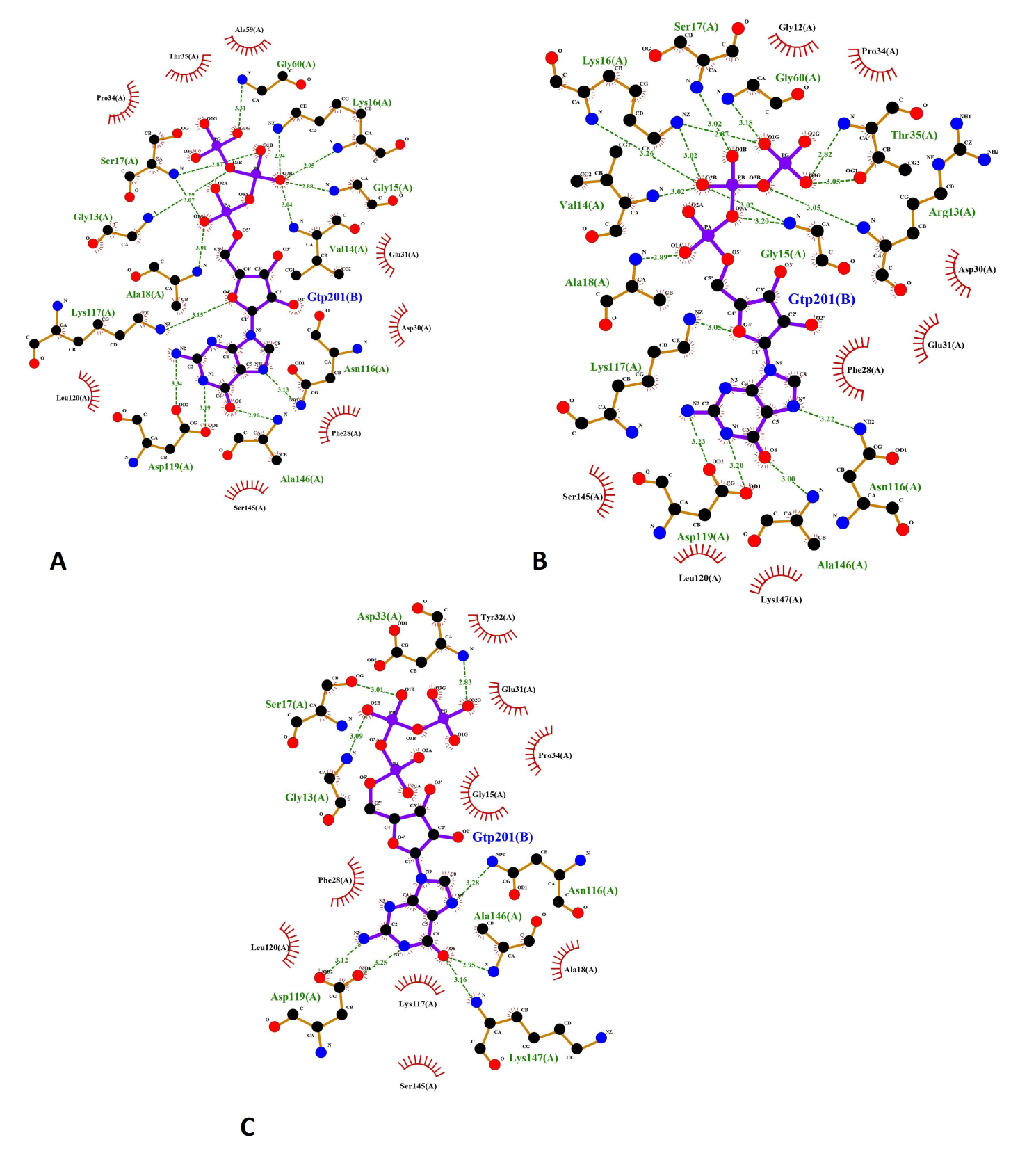


Supplementary figure 8. 2D chart for the reacting residues between GTP and (A) wild NRAS model, (B) mutant model with the highest binding affinity (G13R SNP), and (C) mutant model with the lowest binding affinity (G60E SNP).

**
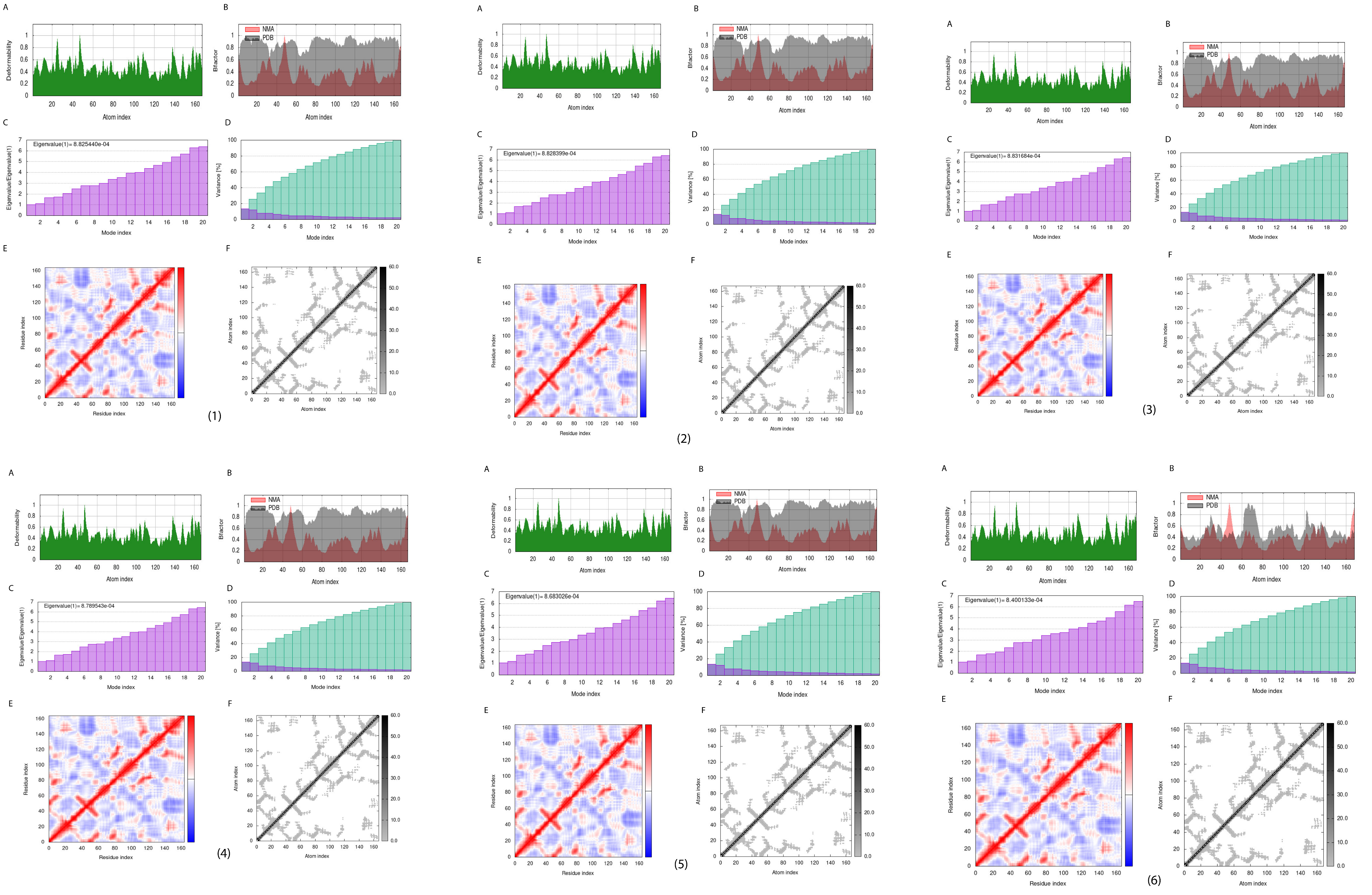
**

**
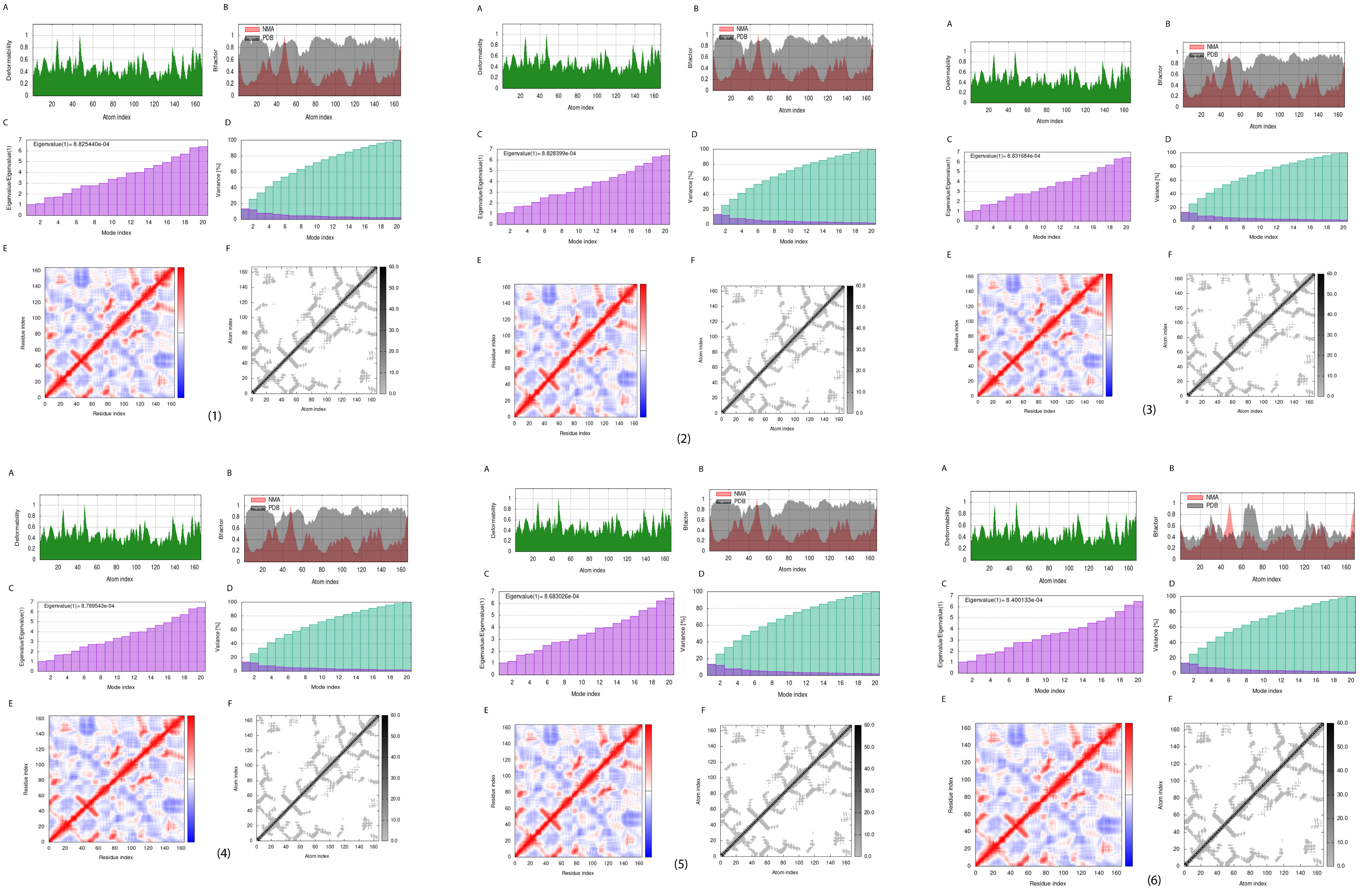
**

**
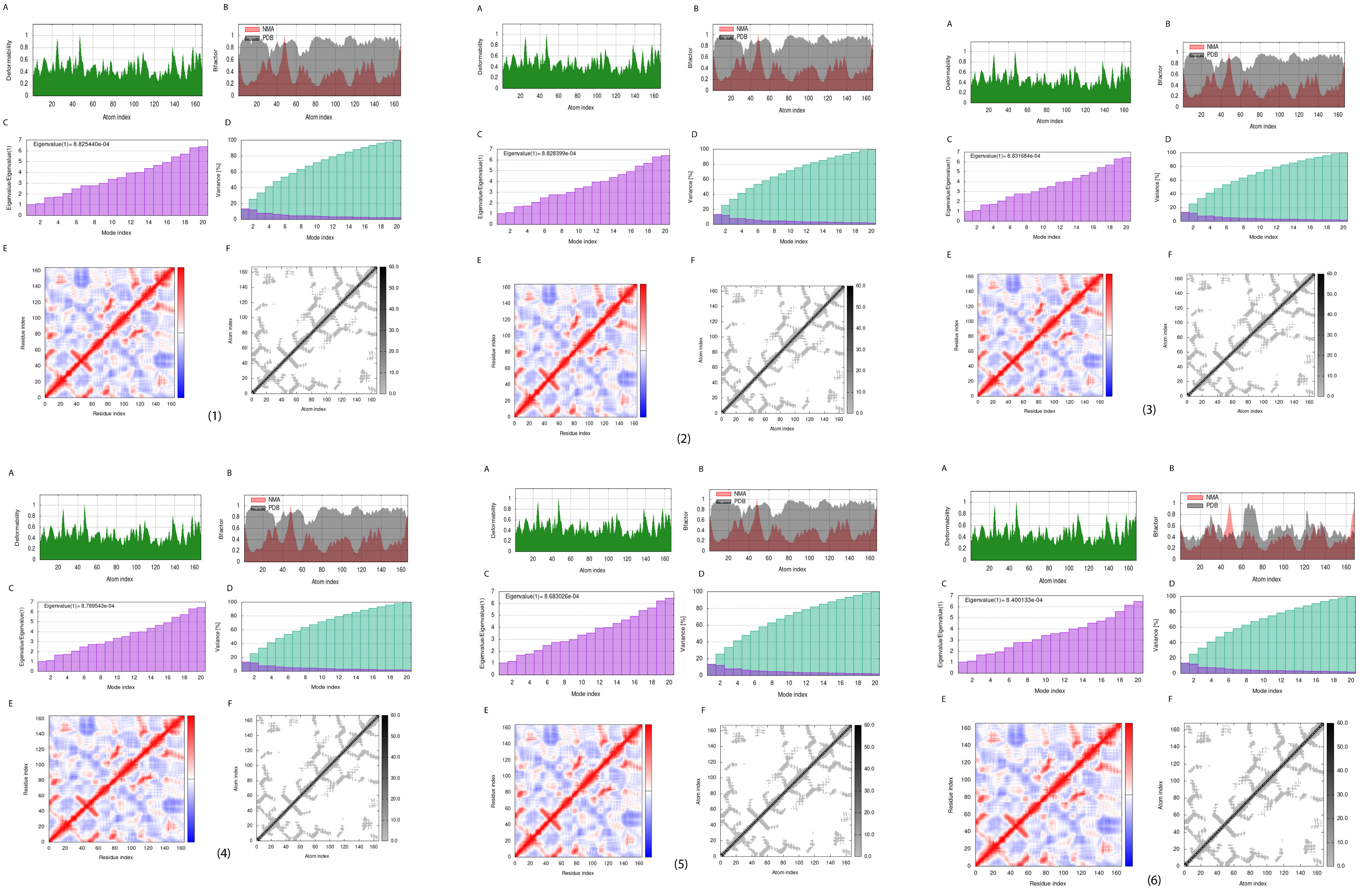
**

**
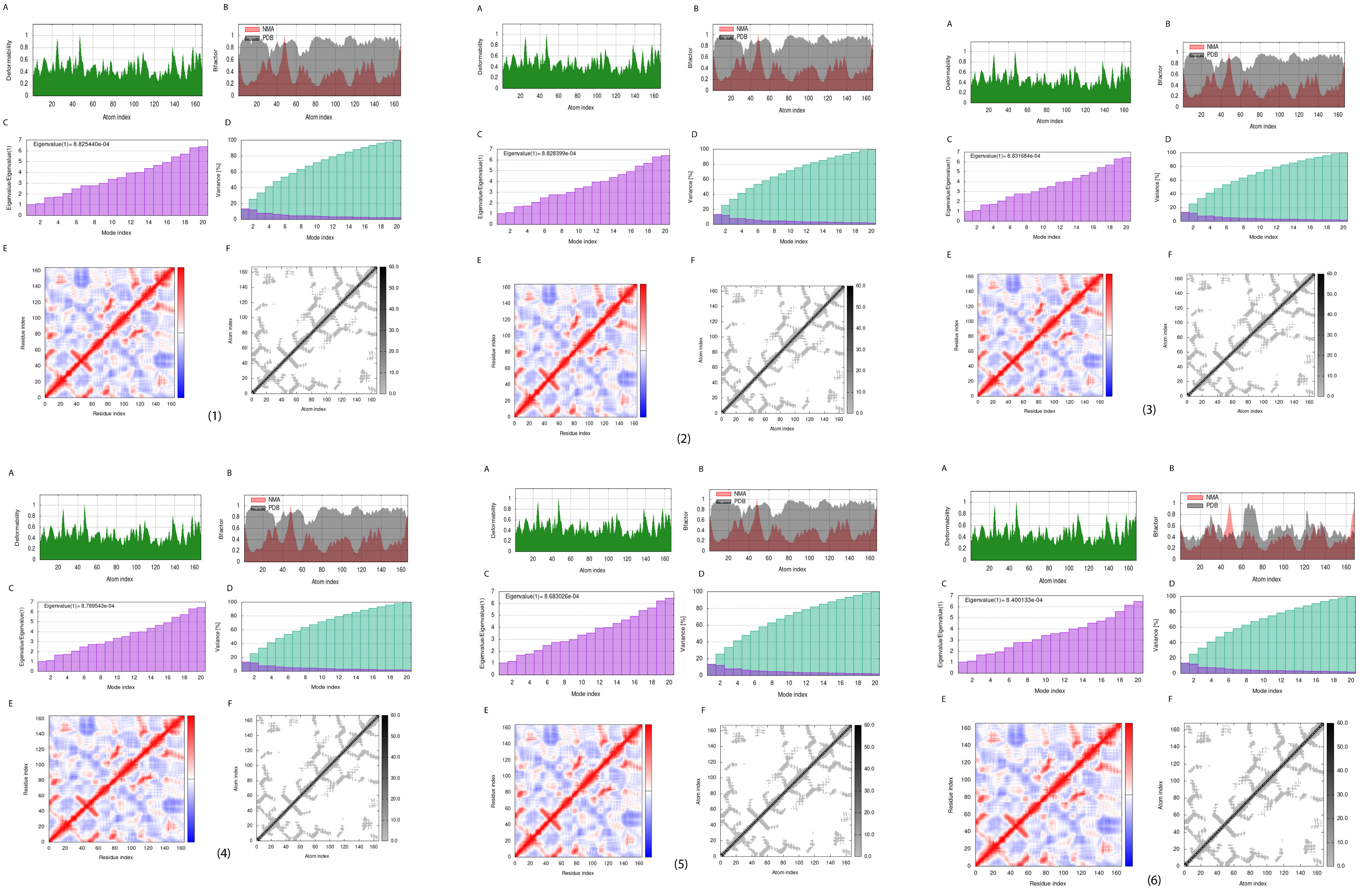
**

**
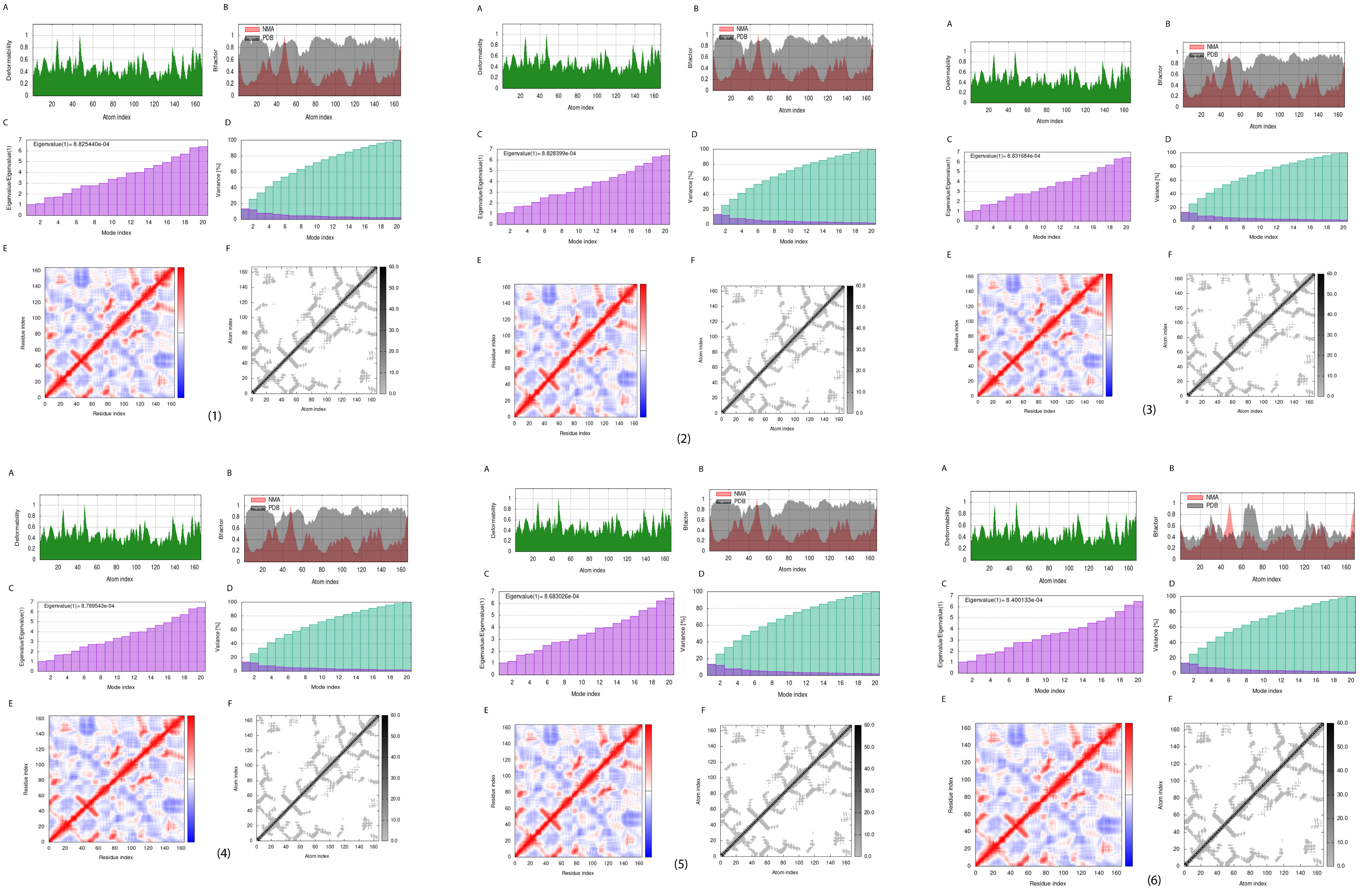
**

**
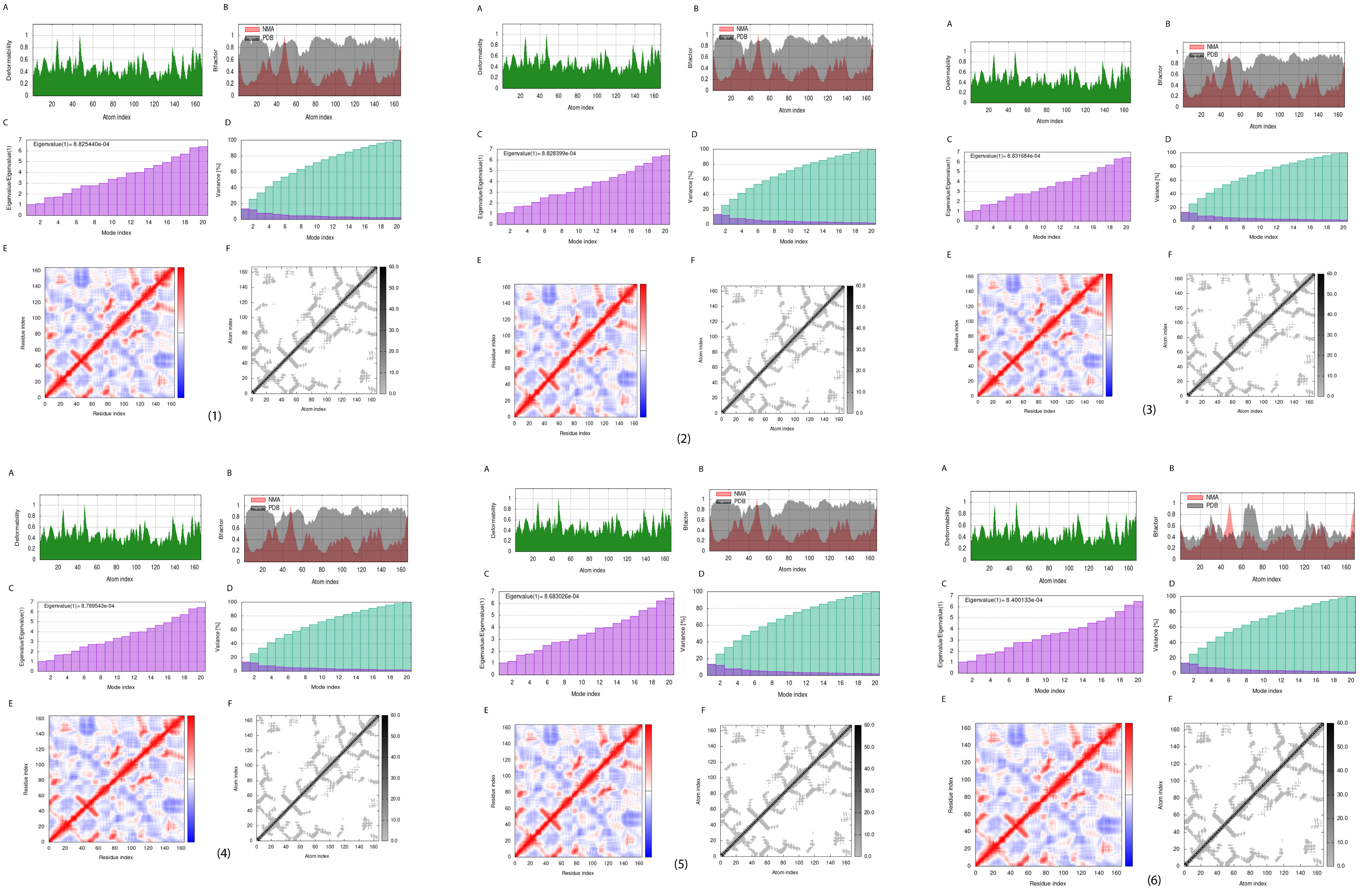
**

**Supplementary figure 9.** Inherited flexibility analysis of the modelled SNP and native GTPase NRAS proteins using normal mode analysis within dihedral coordinates. Complex mobility and flexibility were assessed through (**A**) deformability, (**B**) B-factor values, (**C**) eigenvalue, (**D**) variance, (**E**) covariance of residue index, and (**F**) elastic network analysis for (**1**) SNP model #1, (**2**) SNP model #2, (**3**) SNP model #3; (**4**) SNP model #6; (**5**) SNP model #16; (**6**) native NRAS protein.
